# Emergent Multifunctional Magnetic Proximity in van der Waals Layered Heterostructures

**DOI:** 10.1002/advs.202200186

**Published:** 2022-05-21

**Authors:** Eun‐Mi Choi, Kyung Ik Sim, Kenneth S. Burch, Young Hee Lee

**Affiliations:** ^1^ Center for Integrated Nanostructure Physics, Institute for Basic Science (IBS) Sungkyunkwan University (SKKU) Suwon 16419 Republic of Korea; ^2^ Department of Physics Boston College 140 Commonwealth Ave Chestnut Hill MA 02467‐3804 USA; ^3^ Department of Energy Science Sungkyunkwan University Suwon 16419 Republic of Korea

**Keywords:** 2D vdW materials, heterostructure, magnetic proximity engineering, magnetism, proximity effect, spintronic

## Abstract

Proximity effect, which is the coupling between distinct order parameters across interfaces of heterostructures, has attracted immense interest owing to the customizable multifunctionalities of diverse 3D materials. This facilitates various physical phenomena, such as spin order, charge transfer, spin torque, spin density wave, spin current, skyrmions, and Majorana fermions. These exotic physics play important roles for future spintronic applications. Nevertheless, several fundamental challenges remain for effective applications: unavoidable disorder and lattice mismatch limits in the growth process, short characteristic length of proximity, magnetic fluctuation in ultrathin films, and relatively weak spin–orbit coupling (SOC). Meanwhile, the extensive library of atomically thin, 2D van der Waals (vdW) layered materials, with unique characteristics such as strong SOC, magnetic anisotropy, and ultraclean surfaces, offers many opportunities to tailor versatile and more effective functionalities through proximity effects. Here, this paper focuses on magnetic proximity, i.e., proximitized magnetism and reviews the engineering of magnetism‐related functionalities in 2D vdW layered heterostructures for next‐generation electronic and spintronic devices. The essential factors of magnetism and interfacial engineering induced by magnetic layers are studied. The current limitations and future challenges associated with magnetic proximity‐related physics phenomena in 2D heterostructures are further discussed.

## Graphical abstract

Beyond the conventional magnetic proximity effect, a much broader picture of magnetic proximity effects to control the material functionalities suggests a ubiquitous approach to transform a wide class of materials. Since the discovery of huge library of two‐dimensional materials, magnetic proximity effect is extensively investigated to attain intrinsic spin‐dependent properties from its adjacent for next‐generation electronic and spintronic devices.

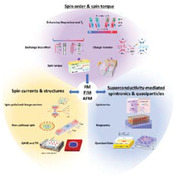

## 1 Introduction

When guest material which possesses long range order is attached to an adjacent host material, proximity effect often occurs in the host. The desired functionalities that are absent from the material can often be generated by the transfer of ordering from the adjacent system. As a result, the proximity effect in the host material enables the manipulation of superconducting, spintronic, excitonic, and topological phenomena. The first demonstration of such an phenomenon was the discovery of superconducting (SC) proximity effect in 1960s, in which the superconducting Cooper pair penetrated into an adjacent metallic layer over a long distance (≈1 µm).^[^ ^1^, ^2^, ^3^ ^]^ Recent interest in superconducting proximity effect was sparked by it being means to superconducting spintronics and Majorana fermions.^[^ ^4^, ^5^ ^]^

In 1973, Zuckermann theoretically predicted the magnetic proximity effect, in which an itinerant ferromagnetic (FM) material could induce ferromagnetism into proximitized paramagnetic metal.^[^ ^6^ ^]^ Over the subsequent several decades, such a conventional magnetic proximity effect has been widely extended to antiferromagnetic insulator in addition to paramagnetic metals. This further elucidates a much broader picture of magnetic proximity effects to control the material functionalities such as magnetic transition temperature (*T*_C_ and antiferromagnetic *T*_N_), coercivity, magnetic anisotropy, and exchange bias effect,^[^ ^7^, ^8^, ^9^, ^10^, ^11^, ^12^, ^13^, ^14^, ^15^, ^16^, ^17^ ^]^ as a ubiquitous approach to transform a wide class of materials. The interplay of magnetic proximity with spin–orbit coupling (SOC) has received interest in 2000s because of its significant potential for spintronics, such as efficient conversion between spin and charge, along with the generation of spin‐polarized currents.^[^ ^18^, ^19^, ^20^ ^]^ Moreover, since the discovery of huge library of 2D materials, magnetic proximity effect has been extensively investigated to attain intrinsic spin‐dependent properties from its adjacent material like magnetic or topological materials. For example, the interfacial charge transfer in atomically thin 2D materials strongly influences the magnetic proximity.

**Figure 1:**
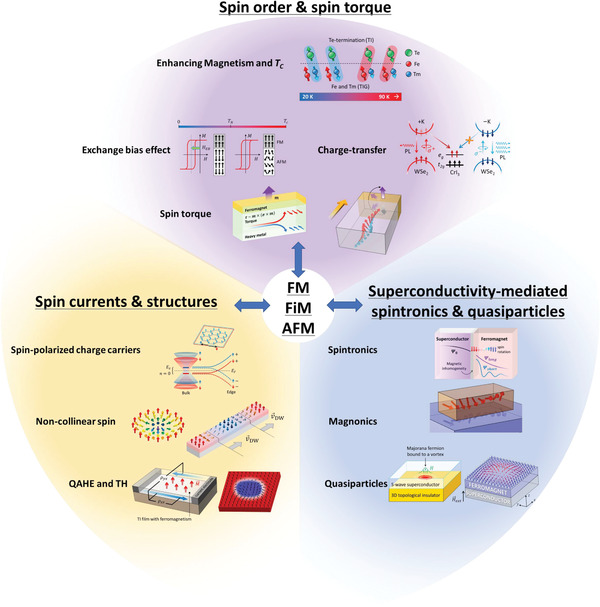
Interfacial phenomena through magnetic proximity. Spin‐control (ordering and torque)‐enhancing magnetism and *T*_C_,^[^ ^9^, ^12^, ^21^ ^]^ exchange bias effect,^[^ ^9^, ^11^, ^17^, ^22^, ^23^, ^24^, ^25^, ^26^, ^27^ ^]^ charge‐transfer,^[^ ^7^, ^28^, ^30^, ^31^, ^32^, ^33^, ^34^, ^35^ ^]^ and spin torque.^[^ ^38^, ^39^, ^102^ ^]^ Control spin currents^[^ ^7^, ^28^, ^30^, ^43^, ^44^, ^48^, ^49^, ^50^, ^51^, ^52^, ^53^, ^54^ ^]^ and (noncollinear) spin structure.^[^ ^55^, ^56^, ^57^, ^58^, ^59^, ^60^, ^61^, ^62^ ^]^ Superconductivity‐mediated spin‐control (spin supercurrent and magnons)^[^ ^4^, ^44^, ^63^ ^]^ and quasiparticle‐generation (Majorana and skyrmions).^[^ ^64^, ^65^, ^66^, ^67^, ^68^ ^]^

The synergistic interfacial engineering of material functionalities through spin statistics and dynamics emerged by magnetic proximity or proximitized magnetism includes i) control of spin and subsequent enhancement of magnetism,^[^ ^9^, ^12^, ^21^ ^]^ exchange bias,^[^ ^7^, ^8^, ^9^, ^11^, ^17^, ^22^, ^23^, ^24^, ^25^, ^26^, ^27^ ^]^ charge transfer,^[^ ^7^, ^28^, ^29^, ^30^, ^31^, ^32^, ^33^, ^34^, ^35^, ^36^, ^37^ ^]^ and spin torque,^[^ ^38^, ^39^, ^40^, ^41^, ^42^ ^]^ ii) control of spin currents^[^ ^7^, ^28^, ^30^, ^43^, ^44^, ^45^, ^46^, ^47^, ^48^, ^49^, ^50^, ^51^, ^52^, ^53^, ^54^ ^]^ and (noncollinear) spin structures in real space,^[^ ^55^, ^56^, ^57^, ^58^, ^59^, ^60^, ^61^, ^62^ ^]^ iii) superconductivity‐mediated control of spin (spin supercurrent and magnons),^[^ ^4^, ^44^, ^63^ ^]^ and iv) generation of quasiparticles such as Majorana and skyrmions^[^ ^40^, ^64^, ^65^, ^66^, ^67^, ^68^, ^69^ ^]^ (**Figure** **1**). These efforts are crucial for forming the “bits” in next generation spintronics, valleytronics, magnonics, and topological quantum computing. Hence, magnetic proximity effect has been extensively investigated for decades in order to control functionalities of proximitized materials.

Nevertheless, the magnetic proximity effect is typically short‐ranged (several nanometers) owing to the finite extension of the electronic wavefunctions across the interface (exchange coupling of spins).^[^ ^6^, ^7^, ^70^ ^]^ Hence, the ability to tune 3D bulk materials via the magnetic proximity effect might be limited, as the host sample size often exceeds the characteristic length of proximity effects (several nanometers).^[^ ^6^, ^7^, ^70^ ^]^ In addition, the proximity effect is optimized at ideal interfaces, i.e., between nearly defect‐free materials. Such ideal interfaces do not generally exist in 3D bulk materials owing to the presence of covalent or ionic bonds; these dangling bonds often generate disorder during the deposition process.^[^ ^71^, ^72^, ^73^, ^74^, ^75^, ^76^ ^]^ The lattice mismatch of ultrathin films of 3D materials might also limit the potential combinations of materials.^[^ ^77^, ^78^, ^79^, ^80^, ^81^, ^82^ ^]^ Therefore, these limitations should be addressed to achieve effective magnetic proximity‐induced phenomena (see **Table** **1**). Moreover, the strong SOC, which is an essential factor for the effective conversion between charge and spin, favors lower dimensions.^[^ ^49^, ^50^, ^83^ ^]^

**Table 1:** Comparisons of heterostructures^[^ ^73^, ^90^, ^93^, ^94^, ^112^, ^113^ ^]^

	3D–3D	2D–3D and 2D–2D
Advantages	Wide range of state‐of‐the‐art devices Strong interfacial coupling between layers Difficulties in deformation Large‐scale fabrication	Atomically sharp interfaces High mechanical flexibility Flexible integration of radically different materials Hybrid‐interface without substrate effect High degree of freedom for tailoring functionalities High magnetocrystalline anisotropy
Drawbacks	Limitation in integration Difficulty in probing interfacial phenomena Thermal magnetic fluctuation Dangling bond Large contact resistance Unexpected interfacial modification Substrate effect High cost facilities	Weak interfacial coupling between layers Interlayer spacing Lateral potential drop Difficulty in controlling orientation and shape Minuscule size of sample
Challenges	Structural optimization Control of defects	Scalable fabrication Sequential chemical synthesis Reliable and addressable contacting to each layer

Unlike 3D materials, 2D van der Waals (vdW) layered materials may offer ultraclean, dangling bond‐free interfaces that prevent undesired interface disorders. vdW materials are easily exfoliated into a few layers (often monolayers) owing to weak vdW interactions.^[^ ^71^, ^72^, ^73^, ^84^, ^85^, ^86^ ^]^ There exists a large library of 2D vdW layered materials.^[^ ^71^, ^79^, ^82^, ^86^, ^87^, ^88^, ^89^, ^90^, ^91^, ^92^, ^93^, ^94^, ^95^, ^96^, ^97^, ^98^ ^]^ Furthermore, the characteristics of these materials, such as giant tunneling magnetoresistance (TMR),^[^ ^99^, ^100^ ^]^ strong SOC^[^ ^7^, ^51^, ^101^, ^102^ ^]^ and efficient voltage control,^[^ ^103^, ^104^, ^105^ ^]^ can overcome the limitations of 3D materials. Thus, vdW layered materials, which feature atomic thicknesses and form atomically sharp interfaces, are an attractive platform to harness the proximity effect. Several recent reviews have explored 2D vdW layered materials, heterostructures and devices.^[^ ^71^, ^76^, ^90^, ^93^, ^94^, ^101^, ^106^ ^]^ However, reviews of the proximity effect induced by magnetic layers and its control on material functionalities are still lacking.

Herein, we provide a comprehensive review of the proximity effect generated by the magnetic (FM, ferrimagnetic (FiM), or antiferromagnetic (AFM)) layer and various induced phenomena that control or enhance the functionalities of 2D vdW layered materials for next‐generation spin‐electronic devices.^[^ ^5^, ^7^, ^11^, ^12^, ^13^, ^14^, ^52^, ^107^ ^]^ We briefly recall the heterostructures and library of 2D vdW layered materials frequently used to host proximity effects. We then describe the characteristic of topological insulators (TIs) to understand the TI‐related phenomena discussed in this review. For an in‐depth understanding, we discuss the intuitive origin of the magnetic proximity effect. We briefly review methods to determine interfacial magnetism and observing proximity‐related phenomena in heterostructures. Finally, we discuss various perspectives of proximity effects to overcome current limitations and enhance exotic physical phenomena.

## 2 Materials for 2D vdW Heterostructures

### 2.1 2D vdW Layered Materials

Since the successful exfoliation of graphene using the “scotch tape technique,” a “wonder of flatland” has emerged in 2D vdW layered materials.^[^ ^71^, ^72^, ^73^, ^84^, ^85^, ^86^ ^]^ This flatland has attracted immense interest as an alternative building block of traditional Si‐based semiconductor devices (Nobel Prize in Physics, 2010).^[^ ^71^, ^72^, ^73^, ^76^, ^84^, ^85^, ^86^, ^95^, ^108^ ^]^ 2D vdW layered materials have been widely explored: metal (graphene), semiconductors (transition metal dichalcogenides (TMDs) and black phosphorus), insulators (hexagonal (h)‐BN), A‐type AFM (FeX_2_ (X = Cl and Br) and CrI_3_), G‐type AFM (MnPX_3_ and X = S or Se), C‐type AFM (MPS_3_ and M = Fe, Co, and Ni), FM (Gr_2_Ge_2_Te_6_, Fe_3_GeTe_2_, MSe_2_ (M = V and Mn), Cr_2_(Si,Ge)_2_Te_6_), helimagnetic/multiferroic (MnI_2_, CoI_2_, and NiX_2_ (X = Br and I)), superconductors (NbSe_2_ and Bi_2_Sr_3−_ *_x_* Ca*_x_* Cu_2_O_8+_ *_y_*) and various topological materials (e.g., monolayer WTe_2_ as TI).^[^ ^71^, ^79^, ^82^, ^86^, ^87^, ^88^, ^89^, ^90^, ^91^, ^92^, ^93^, ^94^, ^95^, ^96^, ^97^, ^98^, ^109^, ^110^ ^]^ The vdW interaction without direct chemical bonds between layers and/or constraints of crystal lattice matching enables the integration of highly disparate materials to create diverse heterostructures with individual functions.^[^ ^94^, ^111^, ^112^ ^]^

2D vdW heterostructures with atomically sharp interfaces and limited interdiffusion of atoms offer a new range of materials for use in a wide array of ultrathin, flexible, and transparent electronic/optoelectronic devices.^[^ ^73^, ^93^, ^94^, ^95^, ^112^, ^113^ ^]^ 2D vdW heterostructures present intriguing possibilities for the generation, confinement, and transport of charge carriers, spins, excitons, photons, and phonons within atomic interfaces, to facilitate the design of unique devices.^[^ ^114^, ^115^, ^116^ ^]^ Emergent phenomena induced at the interfaces of 2D vdW heterostructures include exchange interaction, broken inversion symmetry, charge transfer, interfacial dipole formation, interfacial orbital hybridization, band renormalization, valley‐contrasting physics, electrically tunable interlayer excitons, structural perturbation including strain effects and dielectric screening.^[^ ^36^, ^93^, ^94^, ^99^, ^100^, ^105^, ^112^, ^113^, ^117^, ^118^, ^119^, ^120^ ^]^ These can be applied to tunneling devices, including in‐plane tunneling,^[^ ^114^, ^121^, ^122^ ^]^ vertical field‐effect transistors (FETs),^[^ ^123^ ^]^ memory,^[^ ^124^ ^]^ photodetectors,^[^ ^125^, ^126^ ^]^ solar cells,^[^ ^127^ ^]^ photodiodes,^[^ ^128^ ^]^ and light‐emitting devices.^[^ ^129^ ^]^

Moreover, additional structural degrees of freedom are available in 2D vdW heterostructures, such as material selections with stacking and misorientation angles. The electronic band structures of hybrid artificial materials strongly depend on the number of stacking orders (A–B–C or A–B–A stacking) and twist angles.^[^ ^130^, ^131^, ^132^, ^133^ ^]^ The twist angles between different layers offer another degree of freedom to tailor their electronic properties for exotic physics, as highlighted by the recent observation of correlated insulator behavior and superconductivity in magic‐angle bilayer graphene homostructures.^[^ ^130^, ^131^ ^]^ Additional structural effects are discussed in the section on challenges and perspectives.

The advantages and challenges of 2D vdW heterostructures are summarized in Table 1.^[^ ^73^, ^79^, ^86^, ^88^, ^93^, ^94^, ^95^, ^112^, ^134^ ^]^ Continuous experimentation and prototype development of 2D vdW heterostructures indicate their versatility and practical applications.

### 2.2 Topological Insulator

**Figure 2:**
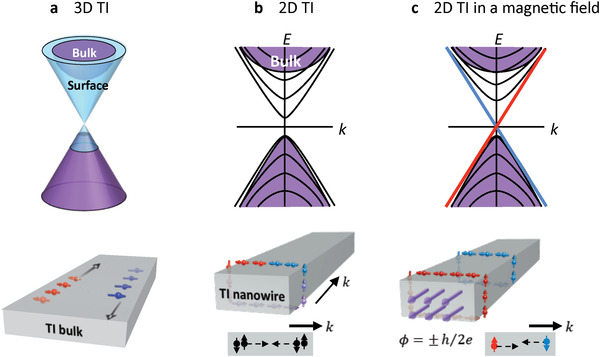
Schematic band structures of topological insulators. a) 3D TI. Spin‐polarized topological surface electrons. b) TI nanowire in a zero‐magnetic field. c) TI nanowire with a magnetic flux *Φ* = ±*h*/2*e*. Reproduced with permission.^[^ ^320^ ^]^ Copyright 2014, ACS Publications.

A TI has a bulk energy gap like an ordinary insulator and topological surface states (i.e., nontrivial metallic states on their surface boundaries), which have linear band dispersion with strong spin‐momentum locking.^[^ ^64^, ^135^, ^136^, ^137^, ^138^, ^139^ ^]^ Due to the bulk‐boundary correspondence, 2D TIs have 1D edge spin‐chiral states and 3D TIs have 2D spin‐helical states (**Figure** **2**). The spin‐momentum locking in these surface states enables one to generate spin‐polarized current under external electric bias, a central feature of TIs in spintronic devices. A quantum anomalous Hall effect, induced by a combination of magnetic polarization and SOC, is a quantized Hall effect that generates a finite Hall voltage even in the absence of an external magnetic field.^[^ ^140^ ^]^ The induced Hall signal can be used to detect skyrmions and further elucidate their motion in devices.^[^ ^141^ ^]^

In addition, nontrivial topological effects result in a non‐Abelian Majorana zero mode. According to the model proposed by Fu and Kane,^[^ ^5^ ^]^ Majorana fermions can appear as Bogoliubov quasiparticles on the surfaces and vortex cores of topological superconductors.^[^ ^142^ ^]^ Based on 2D topological superconductivity, gapless chiral Majorana modes at the boundary of a 2D superconducting TI surface have been proposed in the system of a shallow trench isolation surface overlaid with an FM insulating layer, i.e., a system that synergizes the magnetic and superconducting proximity effect.^[^ ^14^, ^143^ ^]^ In this proposed system, chiral Majorana modes at the edge of the FM layer can be visualized and directly probed using scanning tunneling microscopy (STM) if the magnetic exchange coupling between the FM insulator overlayer and the underlying topological superconducting insulator surface is appropriately tuned. The chirality of the chiral Majorana mode depends on the magnetization direction of the FM domains.

## 3 Fundamentals of Magnetism

**Figure 3:**
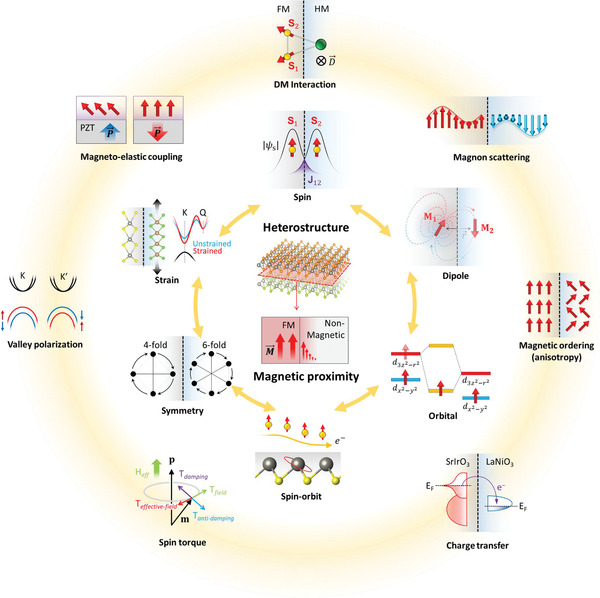
Schematic of physics emerged by magnetic proximity. a) Controllable factors for magnetism: spin, dipole, orbital, SOC (time and spatial inversion) symmetry and strain. b) Interfacial engineering owing to a magnetic layer: Dzyaloshinskii–Moriya interaction (DMI), magnon–magnon scattering, magnetic spin ordering and anisotropy, charge transfer, spin torque, valley polarization/splitting, and magnetoelectric/magnetoelastic coupling.

An in‐depth understanding of the underlying magnetic physics is essential to customize multifunctionality via magnetic proximity. Here, we discuss the key factors—spin exchange, magnetic dipole, orbital occupation, SOC, symmetry and strain (shown in the inner circle of **Figure** **3**)—for the manipulation of magnetism and other related physics. The first four factors are interlinked, which subsequently result in intrinsic magnetism in 3D and 2D magnets. The next two factors, symmetry and strain, have important functions in manipulating interfacial multifunctionalities in heterostructures.

### 3.1 Spin, Dipole, and Orbital

We first discuss the exchange interaction between spins as the most fundamental and influential feature in describing magnetism. An ordered state of spins is generated by the spontaneous breaking of time‐reversal symmetry over macroscopic length scales to yield magnetism.^[^ ^11^, ^90^, ^113^ ^]^ The ordered state of spins is typically driven by the interaction between neighboring spins, i.e., exchange interaction, described by the Heisenberg equation $H\;=\;-\frac{1}{2}\sum_{i,j}J_{ij}S_{i}\cdot S_{j}$, which is fundamentally linked to the overlap of the wavefunctions. The positive exchange interaction in the ferromagnet favors neighboring spins in similar directions to induce magnetic phase transition at a finite temperature. Generally, the exchange interaction stabilizes the magnetic phase in 3D FM materials. In contrast to 3D FM materials, short‐range interaction cannot stabilize long‐range magnetic orders in 1D or 2D FM materials because thermal fluctuations destroy long‐range magnetic orders according to the Mermin–Wagner–Hohenberg theorem,^[^ ^80^ ^]^ so long as the continuous rotational symmetry is not broken by magnetic anisotropy. In 2D vdW layered magnets, a long‐range magnetic order is mediated by intralayer exchange interaction, which has relatively large cation distances owing to the large vdW gap between adjacent layers. This distance weakens the direct exchange interaction. Instead, the superexchange (indirect) interaction or even a super‐superexchange interaction mediated by virtual electron hopping through two anions becomes more dominant.^[^ ^92^, ^144^ ^]^ Because the superexchange interaction is sensitive to the bond length and angle, the strength and sign of the interlayer magnetic order rely on the details of interlayer stacking.^[^ ^144^ ^]^ The most interesting phenomenon induced by (direct or indirect) exchange interaction in magnetic heterostructures is the magnetic exchange field, described as $H\;=\;J\;\frac{\left\langle s\right\rangle }{\mu_{0}\mu_{B}}$, which induces Zeeman splitting, described as *E*_Z_ =  gμ_B_(*B*_ex_ +  μ_0_ *H*), and valley polarization.^[^ ^28^, ^30^, ^31^, ^32^, ^33^ ^]^

Next is a magnetic dipole–dipole (dipolar) interaction between spins that is one of the interactions that describe magnetism. Unlike the short‐range exchange interaction by overlapped wavefunctions, the magnetic dipole interaction is much weaker in the short range because the dipolar coupling constant is proportional to ≈1/*r* ^3^.^[^ ^11^ ^]^ However, over a long range, the magnetic dipole interaction becomes stronger than the exchange interaction, which plays a role in the magnetic order of low‐dimensional FMs such as CrI_3_ and Fe_3_GeTe_2_.^[^ ^11^, ^98^, ^113^, ^145^, ^146^ ^]^

Another basic key factor is an orbital. In bulk magnets, orbital wavefunctions contribute significantly to magnetic energy. In heterostructures, the modification of orbital occupancy (orbital reconstruction) at interfaces can result in novel magnetism alongside electronic states.^[^ ^147^ ^]^ Orbital reconstruction modifies orbital polarization at the interfaces, i.e., change of preferential orbital occupancy (for example, from *d_x_*_2−_ *_y_*_2_ to *d_3z_*_2−_ *_r_*_2_ orbitals under tensile strain), which manipulates the magnetic state in the heterostructure.^[^ ^148^, ^149^, ^150^, ^151^ ^]^ Hence, orbital reconstruction is adopted to explain interfacial magnetism. Two mechanisms of orbital reconstruction have been proposed: i) modulation of the crystal field at the interface, which is a distinct crystal field from the bulk and induces rearrangement of the orbital levels and ii) orbital hybridization to generate covalent bond bridging between orbitals at the interface.^[^ ^147^ ^]^ The hybridization of these orbitals between neighboring atoms results in either metallic or insulating bands, depending on their occupation and the magnitude of electron–electron interactions. Hence, covalent bonding is an important factor in heterostructure engineering.

### 3.2 Spin–Orbit Coupling

SOC is a relativistic effect that occurs because the electric field (due to the positive nucleus) is transformed into a magnetic field (coupled to the electron spin) at a large electron orbital velocity. SOC can split degenerate bands with finite *p*, *d*, and *f* angular momenta and modify the electronic band structure.^[^ ^49^ ^]^ The important functions of large SOCs are i) magnetocrystalline anisotropy through the coupling of electron motion to the crystal electric field, which also stabilizes long‐range magnetic orders in 2D,^[^ ^11^, ^49^, ^98^, ^113^, ^145^, ^146^ ^]^ ii) chiral Dzyaloshinskii–Moriya interaction (DMI), which induces chiral domain walls and skyrmions that are candidates for next‐generation information storage devices,^[^ ^58^, ^59^, ^60^, ^61^, ^62^ ^]^ and iii) effective conversion between charge current and spin current;^[^ ^49^, ^50^ ^]^ for example, the Rashba effect, Rashba–Edelstein effect^[^ ^49^, ^50^, ^152^ ^]^ and spin Hall effect.^[^ ^153^ ^]^ Extremely large SOC materials have been extensively explored to increase the efficiency of functionalities (conversion between spin and charge, generation of spin‐polarized currents, and stabilization of chiral spin structures).^[^ ^49^, ^50^, ^51^ ^]^ 2D systems, such as layered TMDs (WSe_2_ and MoTe_2_) are popular because of their intrinsically significant SOCs (orders of magnitude higher than the SOC in graphene), owing to the presence of heavy elements (such as Mo and W).^[^ ^35^, ^51^, ^101^, ^154^ ^]^ The large SOC observed in 2D TMDs, such as WSe_2_ and MoTe_2_, is the essence of valleytronics because the strong SOC elevate the effective coupling between the spin and valley pseudospin.^[^ ^35^, ^154^, ^155^ ^]^

### 3.3 Symmetry and Strain

Symmetry and strain play important roles to manipulate interfacial multifunctionalities in heterostructures. According to Landau theory, the essence of phase transitions in materials is the change in symmetry; for example, ferromagnetic ordering, which breaks the rotational symmetry in spin space. Here, we discuss the breaking of two symmetries: spatial inversion symmetry (defined as $\vec{r}\to -\vec{r}$) and time‐reversal symmetry (defined as *t* → −*t*).^[^ ^156^ ^]^ The broken symmetry of the order parameters at the interface of the heterostructure results in interesting interfacial phenomena. Broken inversion symmetry induces magnetic anisotropy (resulting in the switching of magnetization),^[^ ^157^, ^158^ ^]^ DMI (control of spin texture, i.e., domain walls and Néel‐type skyrmions),^[^ ^159^, ^160^ ^]^ the Rashba effect and Rashba splitting (spin‐dependent transport phenomena, i.e., conversion between spin and charge).^[^ ^50^, ^107^, ^152^, ^161^, ^162^, ^163^ ^]^ Moreover, a broken time‐reversal symmetry in topological insulators (TIs) generates the quantum anomalous Hall effect state, which is characterized by the quantized Hall conductance (±e^2^/*h*).^[^ ^21^, ^140^ ^]^ Materials that exhibit the quantum anomalous Hall effect are strong candidates for next‐generation spintronic applications because of dissipationless chiral edge current transport in the absence of an external magnetic field and a large and electrically tunable spin polarization.

Material properties can be tuned via mechanical strain or stress engineering, i.e., mechanically shifted structures (bond length, bond angle, and relative positions of atoms). Over recent decades, extensive research on strain engineering in transition metal oxide heterostructures has been conducted to tune the magnetic and electric properties.^[^ ^149^, ^150^, ^151^ ^]^ The transition metal ions at the interface of the heterostructure are surrounded by completely different crystal fields compared to the bulk, creating a network of covalent exchange bonds between metal ion pairs and triggering genuine orbital and spin polarization at the interface of the heterostructure.^[^ ^156^ ^]^ The strain‐driven spin and orbital polarization are typically maintained over a spatial range of tens of nanometers. Like 3D transition metal oxide materials, strain in atomically thin 2D materials can modify their atomic structure, lattice vibration, thermal conductivity, electronic and optical characteristics, electrical and device performance, and chemical activities. Atomically thin 2D materials and their heterostructures are particularly needed to understand strain engineering in terms of bending, wrinkling, stretching, and buckling induced during mechanical exfoliation and transfer.^[^ ^164^, ^165^, ^166^, ^167^ ^]^

## 4 Synergistic Interfacial Engineering Using Magnetic Proximity Effect

Next, we examine the physics at the interface in the heterostructure, which is essential for adjusting functionalities (as shown in the outer circle of Figure 3), DMI, magnon–magnon scattering, magnetic spin ordering and anisotropy, charge transfer, spin torque, and valley polarization or splitting. A brief overview of interfacial engineering via the interplay between two or more key factors of magnetism provides useful concept for tailoring the functionalities of adjacent materials using the proximitized magnetic layers.

### 4.1 Dzyaloshinskii–Moriya Interaction

DMI is an antisymmetric exchange interaction, which involves extending the superexchange interaction to include SOC. Therefore, the strength of the DMI is proportional to the SOC coupling constant. A noncollinear spin ordering induced by DMI indicates the weak FM behavior in the AFM materials^[^ ^58^, ^59^, ^168^ ^]^ and spiral type‐II multiferroicity (coexistence between ferroelectricity and incommensurate magnetism (a spiraling magnetic phase)).^[^ ^169^, ^170^ ^]^ Recently, interesting DMI‐induced interfacial engineering with broken inversion symmetry in the heterostructure is the formation of various noncollinear spin textures, caused by the cross product of spins.^[^ ^58^, ^59^, ^60^, ^61^, ^171^, ^172^, ^173^, ^174^, ^175^, ^176^, ^177^ ^]^ In the FM layer (with perpendicular anisotropy) of FM/heavy metal (with strong SOC) heterostructures, the spins can rotate either along the radius or the circumference to form a vortex configuration, which is a vortex‐ or antivortex‐like magnetization texture protected by a nontrivial topology, i.e., skyrmion.^[^ ^172^, ^173^, ^174^, ^175^ ^]^ DMI‐induced chiral spin structures, particularly chiral domain walls and skyrmions, are relevant for next‐generation information storage devices, including racetrack memory.^[^ ^62^ ^]^

### 4.2 Magnon–Magnon Scattering

Magnons can be used as an alternative to spin or charge currents for logic and memory devices because of advantages such as nanometer wavelength, wide frequency range from GHz to THz, significantly less Joule heating and transmission of spin information in insulators for several micrometers.^[^ ^11^, ^39^, ^178^ ^]^ The information transport using spin waves is called magnonics, like electronics and spintronics. Spin waves are related to the collective excitations of the electron spin system in magnetic metals and insulators. Generally, there are two types of spin waves depending on the coupled electron‐spin interactions, namely, exchange interaction (strong and short‐range, called exchange spin waves) and dipolar interaction (relatively weak and long‐range, called dipolar or magnetostatic waves). While most magnon‐based devices have been based on dipolar spin waves, exchange spin waves have attracted significant interest because of their usage in nm‐sized devices.^[^ ^39^, ^179^ ^]^

Possible applications based on magnons are wave‐based computing, insulator‐based spintronics and data processing over long distances (for example, magnon transistors). Spin waves can be used to change the position of magnetic domain walls through the spin‐transfer torque effect generated from the magnon spin current.^[^ ^180^ ^]^ Various methods have been adopted to manipulate the propagation of spin waves and the properties of magnons;^[^ ^39^ ^]^ for example, control of the geometry of magnetic structures, fabrication of narrow strips and thin films, thermal fluctuations and electrical control exchange bias field or anisotropy in heterostructures. A more comprehensive review of magnonics is elsewhere.^[^ ^178^ ^]^

### 4.3 Magnetic Spin Ordering and Anisotropy

When spins are aligned, there is a preferred orientation. Unlike spins with magnetic isotropy oriented in any direction, spins with magnetic anisotropy are largely restricted to a plane (XY model) or an axis (Ising model) at a finite temperature.^[^ ^11^ ^]^ The magnetic anisotropy is caused by magnetocrystal, shape, stress, and exchange anisotropy.^[^ ^113^ ^]^ Among these, magnetocrystalline anisotropy has a preferred crystallographic direction, i.e., easy‐axis, and is strongly related to the crystal field and SOC.^[^ ^11^ ^]^ Dipolar interactions contribute to the magnetic anisotropy.^[^ ^11^, ^98^, ^113^, ^145^, ^146^ ^]^ Uniaxial magnetic anisotropy (XY model) can stabilize the long‐range magnetic order of planar 2D magnets, resulting in a finite Curie temperature.^[^ ^90^, ^113^ ^]^ In a heterostructure, broken inversion symmetry produces perpendicular magnetic anisotropy that favors magnetization perpendicular to the interface. Perpendicular magnetic anisotropy has been adopted for magnetic recording media owing to its ability to retain a stable magnetization state over a long period.^[^ ^157^, ^158^ ^]^ The interfacial perpendicular magnetic anisotropy can be modulated by applying an electric field normal to the interface. The tuning anisotropy via an applied voltage has potential for the energy‐efficient switching of magnetization in magnetic memories.

### 4.4 Charge Transfer

A key engineering in heterostructure is an interfacial charge transfer through electronic and structural reconstruction at the interface.^[^ ^149^ ^]^ In the heterostructure of semiconductors, a work function mismatch results in an interfacial charge transfer, i.e., equalizing the chemical potentials across the boundary. In complex oxide heterostructures, charge imbalance, differences of chemical potential and band reconstruction at the interface can induce charge transfer across the interface, forming a 2D electron gas and interfacial electronic reconstruction. An intraband charge transfer across interfaces can cause the redistribution of spin and orbital states, which results in interfacial magnetism. This charge transfer‐mediated magnetic coupling is one of the key mechanisms of interfacial magnetism and exchange bias.^[^ ^149^ ^]^ Recently, spin‐polarized charge transfer (hopping) between WSe_2_ (direct‐gap semiconductor) and CrI_3_ (magnetic layer) and the effect on the magnetization of CrI_3_ have been reported, based on valley splitting and intervalley exchange coupling of TMDs induced by a magnetic proximity‐induced exchange field.^[^ ^181^ ^]^ This new concept of charge transfer demonstrates the manipulation of magnetic order through the switching of the individual‐layer magnetization in proximate materials.^[^ ^181^ ^]^ Charge transfer and modulation doping has also been seen using the narrow bands of the magnetic insulator RuCl_3_.^[^ ^36^ ^]^

### 4.5 Spin Torque

An emergent engineering in magnetic‐proximitized heterostructure is switching magnetization by spin torque. The spin current induced by the spin polarization of the charge current produces a magnetic torque, which can be used to manipulate the magnetization by spin‐transfer torque (FM/nonmagnetic metal/FM trilayers or FM/nonmagnetic insulator/FM tunnel junctions) and spin–orbit torque (FM/heavy metal).^[^ ^11^, ^52^, ^182^, ^183^, ^184^ ^]^ The Landau–Lifshitz–Gilbert equation phenomenologically describes the dynamics of magnetization induced by spin torque: $\frac{d\vec{M}}{dt}=\;-r\vec{M}\times {\vec{H}}_{\mathrm{eff}}+\frac{\alpha }{M_{s}}\vec{M}\times \frac{d\vec{M}}{dt}+\frac{\gamma }{M_{s}}\vec{T}$.^[^ ^11^, ^52^ ^]^ The first two terms, $\vec{M}\times {\vec{H}}_{\mathrm{eff}}$ (precession of magnetization) and $\vec{M}\times \frac{d\vec{M}}{dt}$ (Gilbert damping, relaxation of magnetization), describe the dynamics of magnetization without charge current flow. The third term ($\vec{T}$) describes the spin torque when the spin‐polarized current flows, and it consists of two components: damping‐like torque (*T*_DL_) and field‐like torque (*T*_FL_). $\vec{T}$ is determined by the types of spin current, i.e., spin‐transfer torque by a spin‐polarized charge current (spin Hall effect) and spin–orbit torque by a pure spin current (Rashba–Edelstein effect). Recently, spin–orbit torque related to SOC has attracted immense interest because of the higher efficiency of energy and spin‐charge conversion compared to spin‐transfer torque, which requires a high critical current density for switching devices.^[^ ^11^, ^182^, ^183^ ^]^ Another advantage of spin–orbit torque is the nonequilibrium transverse spin currents along the out‐of‐plane direction generated by the in‐plane charge current, which exerts a torque on the FM layer and switches the magnetization.^[^ ^11^, ^182^, ^183^ ^]^ Unlike coupled read and write current paths in spin‐transfer torque devices, decoupled current paths are permitted in spin–orbit torque devices. This enables the improvement of endurance and better read stability in spin–orbit torque devices compared to spin‐transfer torque devices.^[^ ^11^, ^182^, ^183^, ^184^ ^]^

### 4.6 Valley Polarization or Splitting

A valley is a local minimum (maximum) in the conduction (valence) band located away from the Gamma point. The electron valley degree of freedom is the basis of valleytronics.^[^ ^35^ ^]^ The key to valleytronics is the valley‐dependent energy dispersion of carriers, i.e., valley polarization. When the inversion symmetry in the monolayer TMDs (such as WSe_2_, WS_2_, MoSe_2_, and MoS_2_) is broken, the SOC splits the degeneracy between spin up and down, such that the spin and valley are coupled. These degenerate valleys can be split through magnetic proximity‐induced exchange fields in the heterostructure of 2D vdW layered materials with magnetic insulators (e.g., EuS, CrI_3_, or yttrium iron garnet).^[^ ^28^, ^30^, ^31^, ^32^, ^33^, ^34^, ^48^, ^181^, ^185^ ^]^ This is called the valley Zeeman effect.^[^ ^31^ ^]^ These split valleys can be represented by a binary pseudospin that behaves like a spin‐1/2 system; the electron in the +K valley is labelled as valley‐pseudospin up, and the electron in the −K valley is labelled as valley‐pseudospin down.^[^ ^35^, ^154^, ^186^ ^]^ Furthermore, the strong SOC of TMDs results in a coupled spin and valley degree of freedom. This is another important feature of valleytronics.^[^ ^35^, ^154^, ^155^ ^]^ Interestingly, the spin‐aligned configuration causes ultrafast spin‐dependent charge hopping across the interface of the heterostructure. This enables the manipulation of the magnetic order through the switching of individual‐layer magnetization.^[^ ^181^ ^]^ Subsequently, this opens the possibility of operating a valley switch that can serve as a building block for future valleytronic devices via electronic or optical injection and readout.^[^ ^35^ ^]^

### 4.7 Magnetoelectric and Magnetoelastic Coupling

Electric‐field control of magnetism has been a long‐term strategy within the spintronics community. One of possible approaches is magnetoelectric coupling.^[^ ^92^, ^187^ ^]^ Magnetoelectric effect described by Δ*P*  =  *α*_D_Δ*H*, where *P*, *H*, and *α*_D_ define electric polarization, magnetic field, and direct magnetoelectric coupling coefficient, respectively, has been widely investigated in all‐solid state including semiconductor, FM insulator, AFM insulator, and multiferroic material.^[^ ^187^, ^188^ ^]^ The recent discovery of a magnetoelectric effect and strong magnetoelastic coupling in 2D magnet such as CrI_3_, Fe_3_GeTe_2_, and Cr_2_Ge_2_Te_6_ has opened the prospect of the electrical control of magnetism in van der Waals ferromagnetic semiconductors.^[^ ^96^, ^103^, ^105^, ^189^, ^190^, ^191^, ^192^, ^193^ ^]^ Despite all promising demonstration of magnetoelectric coupling in 2D magnet, there are still remaining challenges, i.e., low operating temperature and ionic gating.^[^ ^96^, ^103^, ^105^, ^189^, ^194^ ^]^ Meanwhile, magnetoelasticity and strain‐mediated magnetoelectric effects through ferroelectric or piezoelectric materials (for example, PMN‐PT) have been widely investigated.^[^ ^187^, ^195^, ^196^, ^197^ ^]^ Interestingly, magnetoelasticity enables electric‐field modulation of the magnetic anisotropy, i.e., electrically switching the magnetization orientation, driven by the polarization reversal of the inverse piezoelectric or piezoelastic properties.^[^ ^195^, ^196^, ^198^ ^]^ Recently, 2D vdW heterostructures of FM material and ferroelectric material have been theoretically proposed to achieve switchable ferromagnets and magnetic semiconductors.^[^ ^199^, ^200^ ^]^

## 5 Methods to Investigate Interfacial Phenomena in Heterostructures

Determining interfacial magnetism and observing proximity‐related phenomena in heterostructures are still ongoing challenges because of the short proximity characteristic length, interfacial defect/disorder, minuscule magnetization, and thermal instability of quasiparticles (skyrmion or Majorana fermions).

### 5.1 Determining Interfacial Magnetism

**Figure 4:**
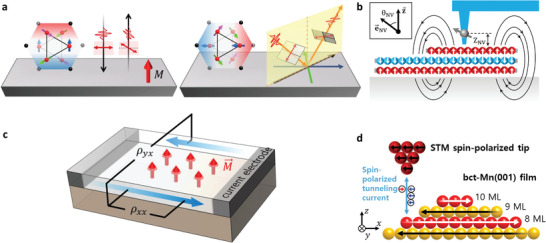
Schematics of representative measurement techniques for interfacial phenomena in heterostructures. a) Polar and longitudinal magneto‐optical Kerr effect.^[^ ^321^ ^]^ b) Scanning single‐spin magnetometry technique. c) Typical Hall effect to probe the anomalous Hall effect. d) Spin‐polarized scanning tunneling microscopy.

Three issues should be considered. First, interfacial magnetism in heterostructures is limited by the characteristic proximity length of ≈2 nm.^[^ ^7^, ^11^, ^70^, ^92^ ^]^ The second issue is whether the magnetic signal is induced by intrinsic magnetic proximity or magnetic defects/disorder that can often be formed in magnetic films and heterostructures during fabrication.^[^ ^11^ ^]^ The third issue is too small volume of 2D materials to provide measurable signals for most conventional magnetic probes, such as vibrating sample magnetometry.^[^ ^92^ ^]^ Polarized neutron scattering and X‐ray magnetic circular dichroism, which have been useful to probe interfacial magnetic ordering and defect effects, are ineffective because of the small dimensions of samples.^[^ ^11^, ^92^, ^144^ ^]^ Therefore, characterization methods are imperative for studying the effects of magnetic proximity in ultrathin materials and interfaces. Recent studies on 2D magnetic materials rely on optical (noncontact in nature) and electrical (electrical leads in contact with the materials) measurements to probe magnetism.^[^ ^7^, ^11^, ^79^, ^92^, ^113^, ^201^, ^202^, ^203^ ^]^ The magneto‐optical Kerr effect is extensively used for such probing. It measures the changes in the polarization rotation angle for linearly polarized incident light. Depending on the light propagation direction with respect to the magnetization direction, it is subdivided into polar and longitudinal measurements depending on the out‐of‐plane and in‐plane magnetization of the material, respectively (**Figure** **4**a).^[^ ^11^, ^92^, ^144^ ^]^ Another optical option is magneto‐optical dichroism to measure changes in the ellipticity of the polarization for reflected or transmitted light.^[^ ^92^ ^]^ Recently, scanning nitrogen‐vacancy (NV) centers in diamond, so‐called NV‐diamond magnetometry, have been attracted much interest as a quantum sensing platform first proposed as magnetic‐field sensors in 2008 (Figure 4b). NV^−^ among three charge states (NV^−^, NV^0^, and NV^+^) exhibits a spin‐1 triplet electronic ground state with a long spin lifetime, and shows a linear Zeeman response for the magnetic field along with spin‐quantization axis ${\vec{e}}_{\mathrm{NV}}$ (Figure 4b).^[^ ^202^, ^203^ ^]^ NV‐diamond magnetometry as a sensitive, atomic‐scale, single‐spin magnetometry enables to quantitatively determine key magnetic properties of 2D materials and to directly image magnetic domains with spatial resolutions of a few tens of nanometers.^[^ ^202^, ^203^ ^]^

With optical approaches, anomalous Hall measurements (electrical transport measurements to probe the spin‐dependent near‐DC conductivities, Figure 4b) is one of the universal probes.^[^ ^92^, ^204^ ^]^ This effect originates from the antisymmetric off‐diagonal elements of the conductivity tensor. In an applied perpendicular magnetic field (*H*_Z_), FM with spin‐polarized free carrier has the total Hall resistance (*R_xy_*) described as *R_XY_* = *R*_0_ *H_Z_* + *R*_S_ *M*_Z_ (*M*_Z_: averaged magnetization), where the first term is the magnetic field‐dependent ordinary Hall effect and the second term is the anomalous Hall contribution. In addition, micro‐Raman spectroscopy and second‐harmonic generation are used to probe the polarization dependence and peak splitting of phonon modes that can reveal magnetism‐induced symmetry breaking. These techniques are unable to detect magnetic order in the absence of strong spin–phonon coupling or symmetry breaking. Despite these limitations, these techniques are useful for detecting AFMs that are inaccessible through the magneto‐optical Kerr effect or dichroism.

### 5.2 Observing Noncollinear Spin States and Majorana Fermions

To detect noncollinear spin states (skyrmions) and Majorana fermions (or zero mode), various approaches (scattering, transport, and imaging) have been adopted, such as neutron scattering, the anomalous Hall effect, fractional Josephson effect, magneto‐optical Kerr effect and spin‐polarized scanning tunneling microscopy/spectroscopy.^[^ ^11^, ^49^, ^61^, ^68^, ^205^, ^206^, ^207^, ^208^, ^209^ ^]^ Among the various approaches, spin‐polarized scanning tunneling microscopy/spectroscopy (Figure 4c) is extensively used to observe spatial maps of nanoscale domains, magnetic ordering and spin texture with atomic resolution.^[^ ^210^ ^]^ Electron tunneling is spin‐dependent because electrons carry spin and charge. Therefore, the tunneling current depends on the magnetic order parameters of both the tip and the sample. Spin‐polarized scanning tunneling microscopy can directly observe complex noncollinear spin configurations in 3D real space with atomic‐scale spatial resolution by combining it with a 3D vector magnet.^[^ ^206^ ^]^ Highly focused spin‐polarized electrons from a sharp spin‐polarized scanning tunneling microscopy tip enable the writing, manipulation, and deletion of individual skyrmions. Moreover, spin‐polarized spectroscopic measurements enable Majorana zero modes to be distinguished from trivial in‐gap states of a superconductor by identifying experimental features that are a direct consequence of the nonlocality of the Hilbert space of Majorana zero modes emerging from a topological band structure.^[^ ^208^ ^]^

## 6 Magnetic Order and Spin Torque

The main aim of the magnetic proximity effect is to control the magnetic order and dynamics of spins in the target materials, which are related to magnetism (magnetization, *T*_C_, *H*_C_) and spin torque.^[^ ^7^, ^11^, ^25^ ^]^ In addition, spin‐dependent charge transfer generated by magnetic exchange fields from adjacent magnetic materials has recently attracted significant interest for valleytronics.^[^ ^28^, ^30^, ^31^, ^32^, ^33^ ^]^ Here, we discuss spin control through magnetic proximity: enhancing magnetism,^[^ ^9^, ^12^, ^21^ ^]^ exchange bias,^[^ ^9^, ^23^, ^24^ ^]^ charge transfer,^[^ ^28^, ^30^, ^31^, ^32^, ^33^ ^]^ and spin torque.^[^ ^38^, ^39^ ^]^

### 6.1 Enhancement of Magnetism toward Magnetic Spintronics and Memory Devices

In heterostructures, at the interface of magnetic (AFM or FM) materials and the adjacent nonmagnetic material, without charge–current flow, the magnetic order parameter (magnetization) significantly penetrates the nonmagnetic region of the adjacent nonmagnetic material with a characteristic length scale of few nanometers (typically ≈2 nm),^[^ ^6^, ^7^, ^70^ ^]^ which is the traditional magnetic proximity effect originating from interfacial exchange coupling. An example is the intrinsic spin polarization of nonmagnetic metals, such as Pt, Ir, and Ta, interfaced with insulating FM layers.^[^ ^211^ ^]^ Recently, the penetration of the magnetic order parameter was adopted to induce magnetism in TI and 2D vdW materials.

We now discuss the proximity‐induced ferromagnetism in TIs (see Section 2.2 for the characteristic of TIs) that produces exotic phenomena such as electric‐field‐induced magnetic monopoles, interfacial magnetoelectric effects, quantum anomalous Hall effect, and Majorana fermions.^[^ ^212^, ^213^, ^214^ ^]^ Among these, the quantum anomalous Hall effect has been extensively studied because the chiral edge current simultaneously carries electrically tunable spin polarization.^[^ ^21^ ^]^ Hence, magnetic TIs can be strong candidates for next‐generation spintronics. Three pathways to achieve magnetic TIs have been pursued: i) dilute magnetic doping such as Cr, V, and Mn,^[^ ^215^, ^216^, ^217^ ^]^ ii) intrinsic magnetic TIs such as MnBi_2_Te_4_,^[^ ^218^, ^219^ ^]^ and iii) magnetic proximity to couple the TI. Despite the early success, dilute magnetic doping induced magnetism in TIs has limitations such as extensive disorder and nontrivial surface states. These can result in defects states in the bandgap of the TIs and the quantum anomalous Hall effect at low temperatures. In addition, the intrinsic magnetic TI MnBi_2_Te_4_ realizes the quantum anomalous Hall effect and axion insulator phases. Even though it is an important breakthrough, the low Néel temperature (≈25 K) prevents noncryogenic applications.

**Figure 5:**
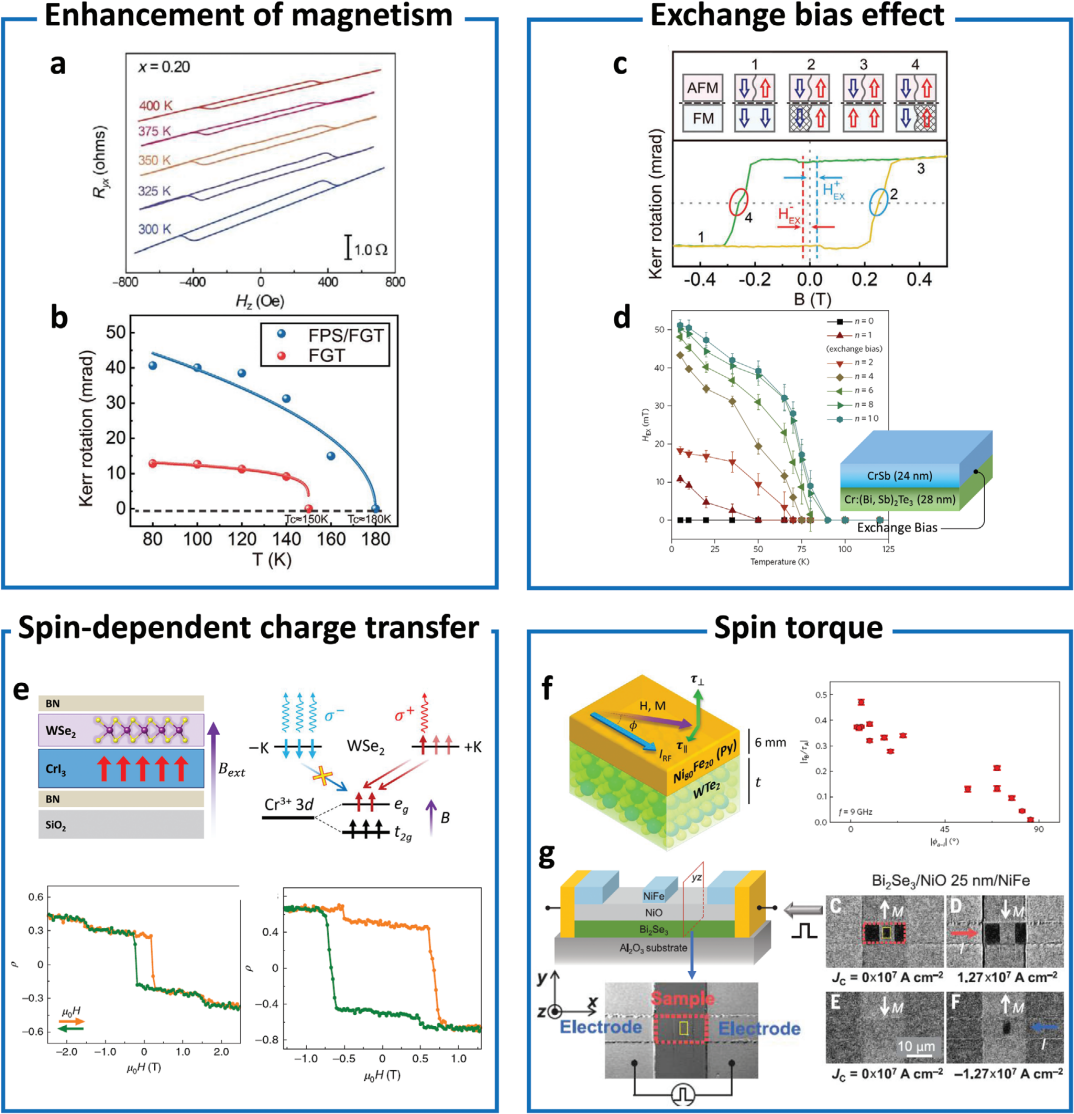
Magnetic order and spin torque. a) Proximity‐induced ferromagnetism above 400 K in an FiM (Tm_3_Fe_5_O_12_)/TI ((Bi*_x_* Sb_1−_ *_x_*)_2_Te_3_) heterostructure confirmed by the anomalous Hall effect for *x* = 0.20 between 300 and 400 K. Reproduced under the terms of the CC‐BY license.^[^ ^21^ ^]^ Copyright 2017, The Authors. Published by AAAS. b) Proximity‐enhanced *T*_C_ in heterostructure of FM (Fe_3_GeTe_2_)/AFM (FePS_3_) confirmed using MOKE. c) Exchange bias effect of FM(Fe_3_GeTe_2_)/AFM (FePS_3_) bilayers confirmed using MOKE. The exchange bias fields are marked by *H* ^+^_EX_ and *H* ^–^_EX_. (b,c) Reproduced with permission.^[^ ^9^ ^]^ Copyright 2020, Wiley‐VCH. d) Reproduced with permission.^[^ ^23^ ^]^ Copyright 2017, Springer Nature Limited. d) Giant enhancements in the exchange field (*H*_EX_) in the superlattices composed of an (AFM (CrSb)/magnetic TI (Cr‐doped (Bi,Sb)_2_Te_3_))*_n_* heterostructure. Reproduced with permission.^[^ ^23^ ^]^ Copyright 2017, Springer Nature Limited. e) Spin‐dependent charge transfer induced by magnetic proximity effect in an FM (CrI_3_)/semiconductor (WSe_2_) heterostructure. Reflective magnetocircular dichroism as a function of magnetic field for a layered AFM trilayer (bottom left) and bilayer (bottom right) CrI_3_. Reproduced under the terms of the CC‐BY license.^[^ ^32^ ^]^ Copyright 2017, The Authors. Published by AAAS. Reproduced with permission.^[^ ^181^ ^]^ Copyright 2020, Springer Nature Limited. f) Control of spin–orbit torques in the heterostructures (left) of TMD (WTe_2_)/FM (Permalloy, Py) by varying the angle of applied current (right). Reproduced with permission.^[^ ^38^ ^]^ Copyright 2017, Springer Nature Limited. g) Magnon‐mediated spin torque in TI (Bi_2_Se_3_)/AFM (NiO)/FM (NiFe)) at room temperature. Reproduced with permission.^[^ ^39^ ^]^ Copyright 2019, AAAS.

Instead, magnetic proximity to couple the TI directly to a high‐*T*_C_ magnetic insulator, such as EuS,^[^ ^12^ ^]^ Y_3_Fe_5_O_12_ (YIG),^[^ ^220^ ^]^ CrSb,^[^ ^23^ ^]^ and Tm_3_Fe_5_O_12_ (TIG)^[^ ^21^ ^]^ can be a good approach to induce magnetism in TIs. In such heterostructures, the squared anomalous Hall effect (see Section 5 for an overview of the measurement technique) provides good evidence of magnetic TIs because of the insulating magnetic layer, i.e., the Hall signal is exclusively from the TIs. Exchange coupling between TI and magnetic insulator enhances the *T*_C_. Recently, a robust FM above 400 K was observed in a TI/TIG heterostructure with a spin‐polarized TI surface (**Figure** **5**a). Such an enhanced *T*_C_ without spin disorder can be the key to invoking the quantum anomalous Hall effect at high temperature. The interdiffusion of magnetization and magnetic exchange coupling can be applied to nonmagnetic 2D vdW layered materials to satisfy the recent demand for 2D magnetic materials, which generates extraordinary spin textures, quantum phases, and quasiparticles.^[^ ^88^, ^113^ ^]^ Moreover, monolayer vdW materials with clean surfaces seem to be ideal host materials for short‐range magnetic proximity effects.^[^ ^11^ ^]^ Proximity‐enhanced ferromagnetism (*T*_C_ improved by more than 30 K and *H*_C_ (Figure 5b), the dots: experimental data, and the solid lines: fitting curves) in 2D vdW heterostructures has been observed using the magneto‐optical Kerr effect (MOKE) (see Section 5).^[^ ^9^ ^]^ This is understood by the proximity exchange coupling.

### 6.2 Exchange Bias Effect toward Magnetoresistive Random Access Memory (MRAM)

In addition to enhancing magnetism, the combination of AFM and FM (top panel of Figure 5c) generates another interesting phenomenon, namely the exchange bias (including switching of magnetization and enhancement of *H*_C_).^[^ ^9^, ^23^ ^]^ The spin configuration of an FM (Fe_3_GeTe_2_) is significantly affected by an AFM (FePS_3_) through proximity coupling in the bilayer. Consequently, both the domain orientations of Fe_3_GeTe_2_ and FePS_3_ at the interface and the exchange bias effect are induced (Figure 5c). The schematic diagram (top of Figure 5c) shows domain of the FM(Fe_3_GeTe_2_)/AFM (FePS_3_) bilayers around interface under different external magnetic fields corresponding to Kerr rotation. In addition to the exchange bias effect in bi‐ or trilayers (AFM/FM or AFM/FM/AFM), *H*_ex_ and *H*_C_ with enhancement of *T*_C_ can be remarkably enhanced in superlattices composed of AFM/FM layers (Figure 5d).^[^ ^23^ ^]^

Our understanding of the exchange bias effect is associated with the exchange interaction at the interface between AFM and FM (or FiM) layers and results in a shift in the magnetic hysteresis (M–H) loops of the FM (or FiM) layers.^[^ ^25^ ^]^ If AFM layer has a large magnetocrystalline anisotropy, the spin order in the AFM layer does not significantly change under a small magnetic field.^[^ ^17^, ^25^ ^]^ Instead, the exchange coupling shifts the M–H loop of the FM layer, which is known as the exchange bias field (*H*_ex_), resulting in unidirectional anisotropy. In the region of low magnetic field, the exchange bias permits magnetization of one FM layer in an FM/AFM heterostructure to be pinned in a fixed direction and magnetization of the other layer to be free to rotate, which creates the spin‐valve structure.^[^ ^221^ ^]^ A spin reorientation of the AFM layer in exchange‐bias FM/AFM heterostructures imparts torque to the AFM layer. This effect is utilized in the read heads of hard disk drives and MRAMs based on spin‐transfer torque.^[^ ^11^, ^221^, ^222^ ^]^ Three factors should be considered to achieve a large *H*_EB_. First, the FM layer should be very thin (typically, a thickness (*t*_FM_) < 20 nm) and strongly magnetic, because *H*_EB_ is proportional to $H_{E}\propto \;\frac{\sqrt{k_{\mathrm{AFM}}}}{t_{\mathrm{FM}}M_{S}},\;$where *k*_AFM_, *t*_FM_, and *M*_S_ are the anisotropy of AFM, thickness of FM, and magnetization, respectively.^[^ ^25^ ^]^ Second, the AFM layer should be considerably thicker than the FM layer to maximize the torque applied to the AFM layer. Third, both FM *T*_C_ and AFM Néel temperature (*T*_N_) should be high, with *T*_C_ > *T*_N_. Moreover, a short spin diffusion length of a few nanometers in the AFM layer should be considered.^[^ ^221^ ^]^ Therefore, vdW layered FM materials could be feasible candidates for hosting the exchange‐bias effect.^[^ ^223^ ^]^

### 6.3 Spin‐Dependent Charge Transfer toward Valleytronics

Another emerging proximity effect is a magnetic exchange bias field that generates a Zeeman splitting (see emergent interfacial engineering via magnetic proximity effect section above and REF^[^ ^30^ ^]^). The Zeeman splitting results in effective valley splitting as the essence of valleytronics is the use of the valley degree of freedom to store and convey information. Valleytronic materials have a band structure composed of two (or more) degenerate but inequivalent valley “states” (local energy extrema) that can be tailored to encode, process, and store information.^[^ ^35^ ^]^ Graphene and monolayer TMDs (e.g., MoS_2_, MoSe_2_, WS_2_, and WSe_2_) provide unique platforms for manipulating valley pseudospins at the band edge. The coexistence of the spin Hall and valley Hall effects in 2D TMDs such as MoS_2_ or WS_2_^[^ ^155^, ^224^ ^]^ presents additional exotic functionalities, such as long‐lived spin relaxation in the nanosecond timescale^[^ ^225^, ^226^ ^]^ and optically/electrically controlled valley polarization.^[^ ^225^, ^227^ ^]^ While valley degeneracy has been lifted, valley splitting in TMDs induced by an external magnetic field is exceedingly small. Such small valley splitting values significantly impede the practical applications and development of valleytronics as magnetic control is almost impossible for small external magnetic fields. A strong interfacial exchange field induced by magnetic proximity could resolve this limitation. An essential key of valleytronics is to tune valley splitting using a large exchange field.^[^ ^28^, ^30^, ^31^, ^32^, ^33^, ^34^, ^181^, ^185^ ^]^

Recently, valley splitting resulting from a magnetic proximity‐induced exchange field in the heterostructure of 2D vdW layered materials (such as graphene or WSe_2_) and magnetic insulators (such as EuS, CrI_3_, or yttrium iron garnet) has attracted interest for application in valley qubits for quantum computing.^[^ ^28^, ^30^, ^31^, ^32^, ^33^, ^34^, ^35^, ^48^, ^154^, ^186^ ^]^ Importantly, the short‐range nature of proximity effects creates a large exchange field (13 T for CrI_3_ and 14 T for EuS),^[^ ^33^ ^]^ mostly within the outermost 2D layer in direct contact with the magnetic material. Recently, several experiments have been performed to manipulate valley‐polarized currents, generate valley polarization and control the energy difference between nearly degenerate valleys.^[^ ^35^, ^186^ ^]^ Moreover, ultrafast spin‐dependent charge hopping across the interface of 2D vdW heterostructures, induced by a large magnetic exchange field (≈13 T), has been reported in FM (CrI_3_)/semiconductor (WSe_2_) heterostructures encapsulated by h‐BN (top panel of Figure 5e).^[^ ^32^, ^181^ ^]^

The effect of spin‐dependent charge transfer between WSe_2_ and CrI_3_ on magnetization was detected using reflective magnetocircular dichroism as a function of the magnetic field (bottom panel of Figure 5e).^[^ ^181^ ^]^ These results demonstrate the manipulation of magnetic order through the switching of the individual‐layer magnetization in the CrI_3_ structure.^[^ ^181^ ^]^ Subsequently, CrI_3_ has attracted much interest because of its potential as a “valley switch.” This type of valley switch can serve as a building block for future valleytronic devices, whether they are based on electronic or optical injection and readout.^[^ ^35^ ^]^

Future applications of valleytronics rely strongly on the Zeeman effect, which splits the energy degeneracy of the two valleys and interfacial interaction with the FM substrate to tune valley splitting.^[^ ^228^ ^]^ The ultrafast charge transfer (hopping) between WSe_2_ and the magnetic layer through valley splitting and intervalley exchange coupling in monolayer TMDs can mediate interactions between layers in different valleys, which can potentially facilitate the engineering of valley‐polarization‐dependent logic gates.^[^ ^35^ ^]^ Despite the significant potential of valleytronics, a short valley lifetime remains a challenge. The valley polarization lifetime should be longer than the gate operation time to realize practical valleytronic devices. In addition, high‐quality FM 2D vdW materials are required for strong magnetic exchange fields, which form the basis of valley splitting.

### 6.4 Spin–Orbit Torque and Magnon‐Mediated Spin‐Transfer Torque toward Spintronics and Magnonics

Effective electrical switching of magnetization from one state to another is essential in the construction of spintronic nanodevices. Here, we discuss two approaches related to the spin current generated by spin–orbit torque and magnon‐medicated spin‐transfer torque.

Current‐induced spin–orbit torque originates from the transfer of orbital angular momentum from the injected carriers to the spin system, such as spin Hall effect and Rashba–Edelstein effect, which results in sustained magnetic oscillations or switching of magnetization.^[^ ^182^ ^]^ Recent studies on spin–orbit torque in the heterostructures of heavy‐metal/FM,^[^ ^229^ ^]^ TMDs/FM,^[^ ^38^ ^]^ and TI/FM^[^ ^102^ ^]^ provide the potential for dramatically improved efficiency in the manipulation of magnetic devices. Recent experimental results show a large out‐of‐plane spin–orbit torque in WTe_2_ arisen from the low crystalline symmetry, i.e., lack of a 180° rotational symmetry around the *c*‐axis (Figure 5f).^[^ ^38^ ^]^ In the bilayer of WTe_2_/Ni_81_Fe_19_ (Permalloy, Py), the charge current flow along the positive (negative) *a*‐axis results in an additional spin–orbit torque around the negative (positive) *c*‐axis. The in‐plane spin–orbit torque is proportional to the angle between the magnetization and in‐plane spin polarization owing to the charge current generated by the spin Hall effect and/or the Edelstein effect. The dependence of the measured torques on the WTe_2_ thickness provides insight into the mechanism of torque generation. The thickness‐independent behavior of the torque ratio implies that the torque is generated by the interface effects. These results present the possibility of generating efficient spin–orbit torques based on broken crystal symmetry and significant SOC. A large spin–orbit torque was achieved for a TI/FM heterostructure owing to the quantum confinement effect with charge‐to‐spin conversion efficiency tuned by the size and dimensionality of the TI.^[^ ^102^ ^]^ Interestingly, FM switching was observed at room temperature with a low critical magnetization‐switching current density. This indicates that the spin‐momentum locking properties of TIs are very useful for magnetization switching via the spin–orbit torque.^[^ ^102^, ^230^ ^]^ Progress has been achieved in generating spin current from spin–orbit torques in different TI materials.^[^ ^231^, ^232^, ^233^ ^]^

In addition to the related spin currents, recent experimental results on magnon current facilitate energy‐efficient control in spintronic devices.^[^ ^39^, ^178^ ^]^ The electrical spin current is generally associated with electron spin and charge flow, which induce Joule heat and power dissipation. The propagation length (diffusion length) of the spin current is relatively short (≈ nm), which prevents the delivery of spin information over long distances.^[^ ^234^ ^]^ By contrast, the magnon current carries the spin angular momentum by the precession of spin moments rather than moving electrons.^[^ ^178^ ^]^ Hence, the magnon torque associated with the magnon current (no electron movement) has less Joule heat dissipation. In addition, a magnon current can flow in an insulator for several micrometers; thus, materials for magnon torque are not necessarily electrically conducting. Such concepts for magnon‐torque‐induced magnetization switching have been demonstrated in heterostructures of TI/AFM/FM devices at room temperature (Figure 5g).^[^ ^39^ ^]^ Magnon‐torque‐driven magnetization switching in a device by injecting a pulsed current I along +*x* axis (upper two images) or −*x* axis (lower two images) is observed by MOKE (right of Figure 5g). The magnon currents carry spin angular momentum efficiently without moving electrons through the AFM insulator. The magnon torque is sufficient to control the magnetization. This represents the possibility of magnon‐based memory and logic devices, i.e., magnonics.

### 6.5 Challenges

To improve the performance of spintronic devices, several challenges remain to be resolved: i) electric‐tunable magnetism at room temperature,^[^ ^87^, ^108^, ^113^, ^235^ ^]^ ii) strong exchange‐bias effect at room temperature, iii) sizable valley splitting and valley polarization with high efficiency and longer lifetimes,^[^ ^236^ ^]^ and iv) effective spin current generation.^[^ ^201^ ^]^ Strong SOC materials, gate‐tunable magnetic proximity and the control of spin scattering at the interface should be explored.^[^ ^47^, ^52^ ^]^ In addition, for valleytronics, the introduction of 2D vdW FM materials is essential because the strength of the magnetic exchange field (the basis of valley splitting) depends critically on the interface and the properties (surface or Curie temperature) of the FM layer.

## 7 Spin‐Current and Noncollinear Spin‐Structure

Since the discovery of giant magnetoresistance (GMR) in the 1980s,^[^ ^237^ ^]^ the spin current has historically referred to the flow of electrons carrying spin information. Over the last few decades, intensive research has been performed on the interplay between spin and other degrees of freedom.^[^ ^43^, ^238^ ^]^ The control of spin currents (accumulation, transport, and detection) is an essential component of spintronics because spin torque (both spin–orbit and spin‐transfer) is generated by spin current.^[^ ^238^ ^]^ In addition, noncollinear spin structures such as skyrmions and magnetic domain walls are strong candidates for next‐generation information storage or logic devices such as racetrack memory and skyrmion‐based magnetic computation.^[^ ^62^, ^239^ ^]^ Hence, controlling the spin current and spin structure is highly demanding for next‐generation spintronics. In this section, we discuss effective approaches to control the spin current and structures through the magnetic proximity effect.

### 7.1 Control of Spin Current toward Spintronics

**Figure 6:**
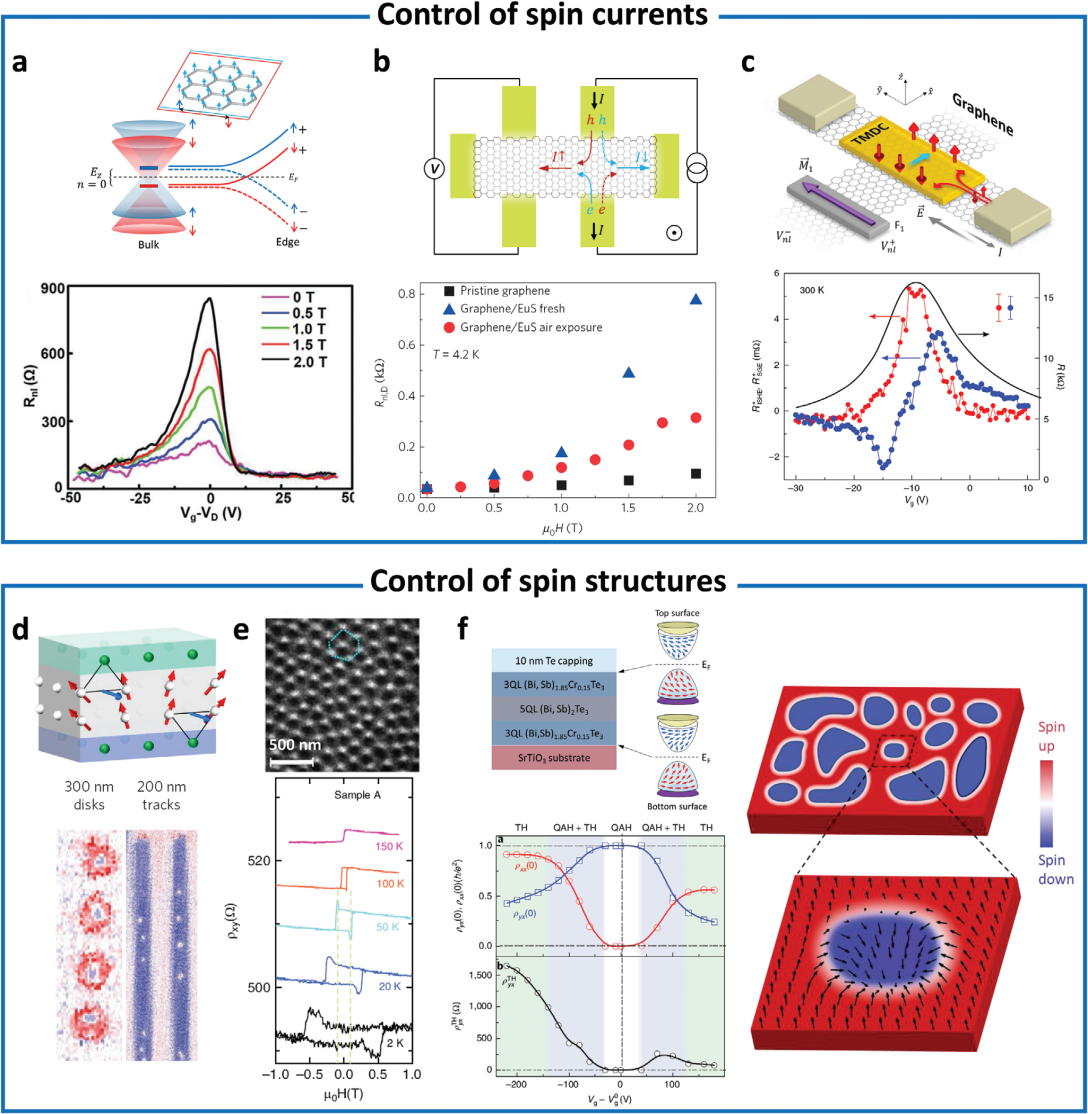
Spin current and noncollinear spin texture. a) Efficient spin injection through the magnetic proximity effect. Reproduced with permission.^[^ ^240^ ^]^ Copyright 2020, Wiley‐VCH. b) Spin‐current generation in the FM (EuS)/metal (graphene) heterostructure. Reproduced with permission.^[^ ^30^ ^]^ Copyright 2016, Springer Nature Limited. c) Proximity‐induced spin‐to‐charge interconversion in graphene/TMD heterostructures as a function of back‐gate voltage (*V*_g_) at room temperature. Reproduced with permission.^[^ ^241^ ^]^ Copyright 2020, Springer Nature Limited. d) Stabilization of small individual skyrmions in an asymmetric (Ir/Co/Pt)_10_ multilayer observed scanning transmission X‐ray microscopy at room temperature and 80 Oe. Reproduced with permission.^[^ ^244^ ^]^ Copyright 2016, Springer Nature Limited. e) Formation of Néel‐type skyrmion in WTe_2_/Fe_3_GeTe_2_ at 180 K with a field of 510 Oe observed using Lorentz transmission electron microscopy (top) and Hall resistivity to show a sign of the topological Hall effect. Reproduced under the terms of the CC‐BY license.^[^ ^69^ ^]^ Copyright 2020, The Authors. Published by Springer Nature Limited. f) Coexistence of chiral edge states and spin textures. Reproduced with permission.^[^ ^245^ ^]^ Copyright 2020, Springer Nature Limited.

Here, we discuss the control (generation) of spin current in terms of effective spin injection and spin‐to‐charge interconversion. An efficient spin injection is a prerequisite for spintronic devices. Several approaches are available to induce the spin, such as conducting ferromagnetic electrodes, magnetic dopants, and magnetic exchange interaction induced by magnetic proximity effects. Among these, magnetic proximity is an effective method without restricting the design of spin switches or degradation of properties. Zeeman splitting of the Dirac cone by a magnetic exchange field from the magnetic layer induces spin‐up hole‐like and spin‐down electron‐like carriers at the charge neutrality point (top panel of **Figure** **6**a).^[^ ^30^ ^]^ Under a Lorentz force, these electrons and holes propagate in opposite directions, resulting in a pure spin current and nonlocal voltage (*V*_nl_). Hence, applying an external magnetic field to this system generates a pure transverse spin current, namely the Zeeman spin Hall effect. In a finite field, the large nonlocal resistance (*R*_nl_) peak near the bias voltage (back‐gate voltage *V*_g_) corresponding to the Dirac point (*V*_D_), *R*_nl,D_, is the signature of the Zeeman spin Hall effect (spin current) (bottom panel of Figure 6a). The comparison of *R*_nl,D_ with and without a magnetic layer (EuS) at *V*_g_ (back gate) = *V*_D_ (the Dirac point) clearly indicates the effective spin injection (generating pure spin current) through the magnetic proximity effect (Figure 6b).^[^ ^240^ ^]^ The driven current produces transverse spin up (red) and spin down (blue) currents under an external perpendicular magnetic field (top panel of Figure 6b).

The proximity‐induced spin–orbit interaction by TMDs results in gate‐tunable spin‐to‐charge interconversion in graphene, even at room temperature (Figure 6c).^[^ ^241^ ^]^ The main elements of the device include the graphene Hall device cross the TMD strip over one of the arms and ferromagnet (F1) contacting the other as ferromagnetic spin injector (top panel of Figure 6c). Gate‐tunable inverse spin Hall and spin galvanic effects are produced because spin‐to‐charge interconversion is driven by the (inverse) spin Hall (red, ISPH) effect and inverse spin galvanic effect (blue, SGE) (bottom panel of Figure 6c). The charge current generates a transverse spin current and nonequilibrium spin density (which can manipulate the magnetization) through the spin Hall and inverse spin galvanic effects, respectively.^[^ ^153^, ^242^ ^]^ Their inverse effects convert the spin current (inverse spin Hall effect) and spin densities (spin galvanic effect) into charge current. The (inverse) spin Hall and (inverse) spin galvanic effects are used for magnetic memory (spin Hall and inverse spin galvanic effects) and logic technologies (inverse spin Hall and spin galvanic effects). This presents the possibility of electric‐tunable spin generation and building blocks for ultracompact magnetic memory technologies.

### 7.2 Noncollinear Spin Structure toward Skyrmions and Magnetic Domain Walls

Noncollinear (chiral) spin structures such as chiral domain walls and magnetic skyrmions induced by DMI (top panel of Figure 6d) have attracted intense interest because of their potential applications in high‐density and energy‐efficient memories.^[^ ^206^, ^243^ ^]^ Several applications and architectures have been proposed and modeled, including skyrmion‐based memory devices analogous to domain‐wall‐based racetrack memory devices.^[^ ^206^, ^243^ ^]^ The stabilization of exotic noncollinear spin structures at RT is still challenging. Here, we discuss representative results in three distinct systems: i) multilayers of heavy metals and FM,^[^ ^244^ ^]^ ii) bilayers of 2D vdW FM and strong SOC materials,^[^ ^69^ ^]^ and iii) trilayers of magnetic‐TI/TI/magnetic‐TI.^[^ ^245^ ^]^

The use of multilayered materials combining interface‐driven out‐of‐plane magnetic anisotropy and additive DMI at successive interfaces is an effective approach of generating skyrmions because of the additive chiral interaction at interfaces (Figure 6d).^[^ ^244^ ^]^ Repetitions of the heavy metal A (blue)/FM (gray)/heavy metal B (green) layers stabilize the skyrmions against thermal fluctuations. In successive Co layers, skyrmions are coupled through ultrathin nonmagnetic layers. Columns of the coupled skyrmions with large magnetic volumes can stabilize them at room temperature, even when the diameter of the column is small. Moreover, such interface‐induced skyrmions with a sub‐100 nm size can be reduced even further by tuning the magnetic anisotropy. Evolution of the skyrmion in the patterned nanoscale disks (300 nm diameter) and tracks (200 nm wide tracks) has been observed by scanning transmission X‐ray microscopy at 80 Oe (bottom panel of Figure 6d). Néel‐type skyrmion lattices and stripe‐like magnetic domain structures with the topological Hall effect were observed in a 2D vdW heterostructure of WTe_2_/Fe_3_GeTe_2_ (Figure 6e), where the DMI was largely enhanced by the interfacial Te atoms (WTe_2_ and Fe_3_GeTe_2_) coupling. A large DMI is significant for generating skyrmions.^[^ ^69^ ^]^ In addition, the strong spin–orbit interaction from WTe_2_ reorganizes the spin polarizations in Fe_3_GeTe_2_, which forms the magnetic domain. The heterostructures of TIs can mediate the interfacial DMI as a consequence of broken inversion symmetry and strong SOC, which stabilizes chiral Néel‐type spin textures.^[^ ^159^, ^160^ ^]^ In addition, the quantum anomalous Hall effect related to the chiral edge state has been observed in magnetic TIs. Both effects have a common prerequisite, i.e., time reversal symmetry breaking. However, quantum anomalous Hall and topological Hall effects have been separately observed in magnetic TIs with distinctly different sample geometries, insulating regimes and metallic systems. Moreover, the topological Hall effect (chiral spin structure) requires a large DMI. Recently, an interplay between chiral edge states and chiral spin structures in magnetic TI sandwich heterostructures was reported (Figure 6f).^[^ ^245^ ^]^ Top left panel shows a schematic of the magnetic topological sandwich heterostructure. This coexistence of chiral edge states and spin textures is confirmed by the concurrence of quantum anomalous and topological Hall effects (Hall resistance *ρ_yx_* (empty blue squares), longitudinal resistance *ρ_xx_* (empty red circles), and topological Hall resistance *ρ_xx_* ^TH^ (empty black circle)) by applying a gate bias at 0 Oe and 30 mK (left bottom panel of Figure 6f). The region of the coexistence of quantum anomalous Hall and topological Hall effects is shaded light blue. The right of Figure 6f shows the formation of the chiral domain walls during magnetization reversals. This indicates a new pathway for spintronics, in which the chiral spin texture associated with the topological Hall effect can be used to record the spin information, which can be transferred through the chiral edge channels of the quantum anomalous Hall effect.

### 7.3 Challenges

Several challenges remain in the generation/detection of spin current, new spin structures with high stability in trivial spin structures and very fast domain wall motion for fast switching: i) strengthening the SOC,^[^ ^246^, ^247^, ^248^, ^249^, ^250^, ^251^ ^]^ ii) improving the spin‐to‐charge conversion efficiency at room temperature,^[^ ^205^, ^241^, ^246^, ^250^ ^]^ and iii) realizing the room‐temperature stabilization of small individual skyrmions for ultradense data storage.^[^ ^49^, ^61^, ^206^ ^]^ The strength of the SOC can be tuned by various material options depending on the design requirements.^[^ ^246^ ^]^ Various combinations of AFM or FiM materials and designs with optimum anisotropy and minimal layer thicknesses can increase thermal stability and promote low switching‐current densities. For skyrmion formation, the Moiré pattern, interlayer and magnetic coupling in vdW heterostructures of 2D magnets (M monolayer on layered AFM substrate with lateral Néel order) can be effective.^[^ ^252^ ^]^

## 8 Superconductivity‐Related Spintronics and Quasiparticles

For effective interconversion between spin and charge currents, numerous interesting quantum materials with special spin interactions, such as 2D TMDs, graphene, TIs and Weyl semimetals have been extensively investigated.^[^ ^44^ ^]^ Beyond conventional electron‐mediated spin currents, spin‐polarized supercurrents can be mediated and controlled using spin‐triplet pairs that can be generated at the magnetically inhomogeneous interface of the superconductor and FM.^[^ ^4^, ^44^, ^253^ ^]^ Another novelty of the magnetic proximity effect on superconductors is the creation of Majorana fermions for fault‐tolerant topological quantum computing aided by their non‐Abelian characteristics.^[^ ^254^, ^255^, ^256^ ^]^ Recently, theoretical studies on heterostructures of superconductors and FM have demonstrated that the stray magnetic field of superconducting vortices may be able to create magnetic skyrmions in the ferromagnetic layer.^[^ ^257^ ^]^ In this section, we discuss magnetic proximity‐mediated spin supercurrents^[^ ^258^ ^]^ and magnons^[^ ^63^, ^259^ ^]^ for spintronics and magnonics and the generation of superconducting quasiparticles such as Majorana^[^ ^66^, ^209^, ^260^ ^]^ and skyrmions.^[^ ^257^, ^261^, ^262^ ^]^

### 8.1 Control of Spin‐Triplet Supercurrents toward Superconducting Spintronics and Magnonics

**Figure 7:**
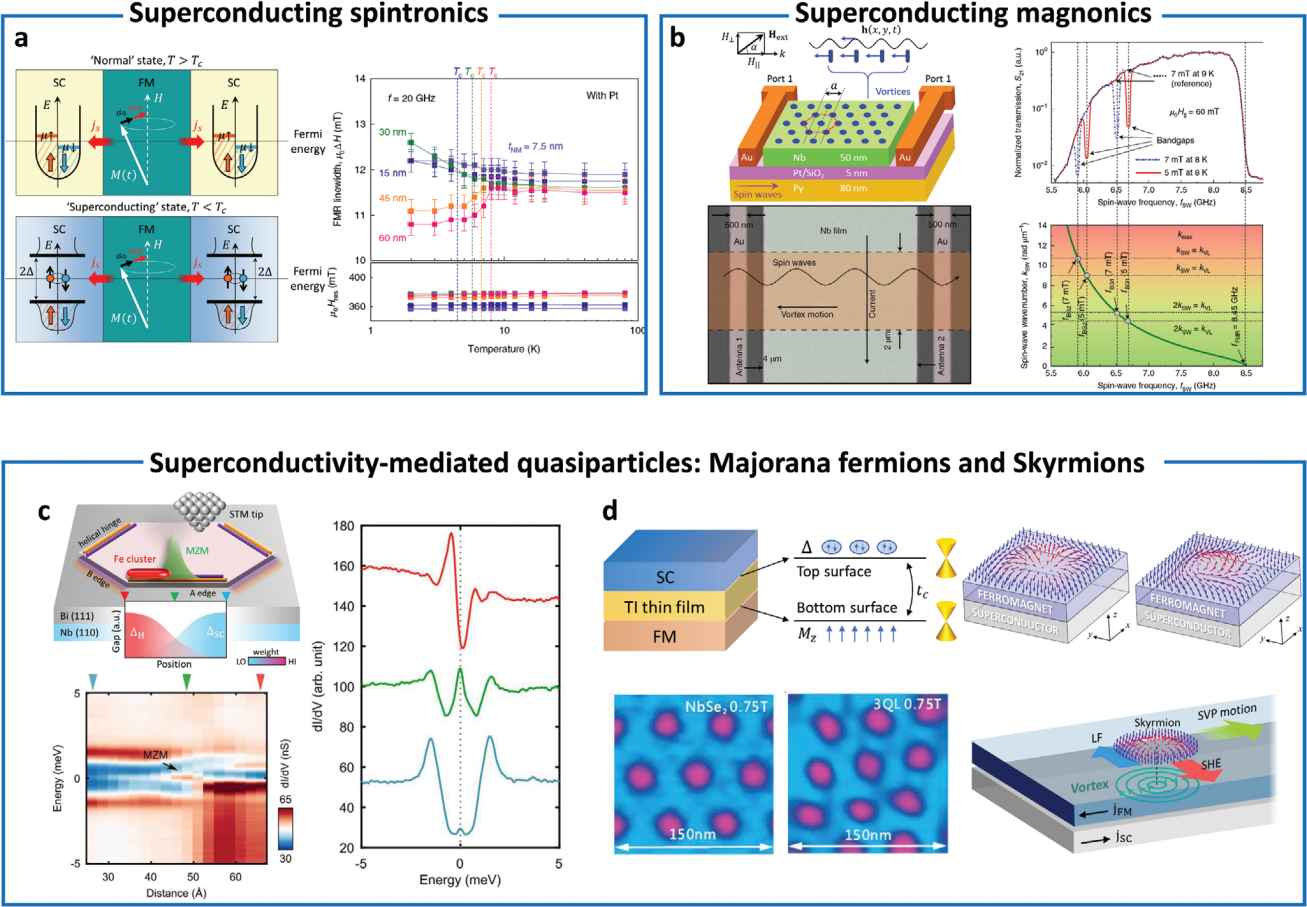
Superconductivity‐related spintronics and quasiparticles. a) Generating superconducting spin currents. Schematic diagrams of the magnetization dynamics and the resulting spin transport in a symmetric FM/SC/FM structure above *T*_C_ (top left) and below *T*_C_ (bottom left). Temperature dependence of the FMR linewidth (top right) and the resonance magnetic field (bottom right) for various Nb thicknesses. Reproduced with permission.^[^ ^258^ ^]^ Copyright 2018, Springer Nature Limited. b) Interaction of spin waves with a flux lattice in heterostructure of FM and superconductor. Schematics of experimental system (top left) and false‐color scanning electron image (bottom left) of the Nb and Au layout. Fluxon (Abrikosov vortices, i.e., the formation of vortices of supercurrent)‐induced magnonic (right). Reproduced with permission.^[^ ^259^ ^]^ Copyright 2019, Springer Nature Limited. c) Majorana zero modes at the interface between FM cluster and superconducting helical edge channel. Schematic diagram (top left). A sharp zero bias peak (ZBP) within the pairing gap along the Bi bilayer (bottom left). Individual point spectra at locations indicated by triangular markers (right). Reproduced with permission.^[^ ^209^ ^]^ Copyright 2019, AAAS. d) Superconductivity‐mediated quasiparticles. A heterostructure of FM/TI/SC for generating Majorana zero modes (top left). Zero‐bias d*I*/d*V* maps measured at 0.4 K and 0.75 T for NbSe_2_ and three quintuple layers (QL) Bi_2_Te_3_/NbSe_2_ heterostructures (bottom left). Skyrmion‐induced bound state (top right). Manipulation of magnetic skyrmion by superconducting vortices (bottom right). Top left: Reproduced with permission.^[^ ^279^ ^]^ Copyright 2019, Springer Nature. Bottom right: Reproduced with permission.^[^ ^257^ ^]^ Copyright 2019, American Physical Society. Bottom left: Reproduced with permission.^[^ ^261^ ^]^ Copyright 2014, American Physical Society. Top right: Reproduced with permission.^[^ ^262^ ^]^ Copyright 2016, American Physical Society.

Generally, the electron orders interfere destructively at the interface between the superconductor and FM layer. However, under the right conditions, superconductivity and spin polarization can unite to create a new state for spin transport in which Joule heating and dissipation are minimized.^[^ ^4^ ^]^ A spin‐triplet state is generated at carefully engineered superconductor interfaces with a FM. This is the basis for superconducting spintronics. Magnetization dynamics generate a spin angular momentum flow into adjacent materials (spin pumping). This is coupled with the spin dynamics of the superconductor via interfacial s–d exchange interactions between conduction electrons and localized magnetic ions.^[^ ^258^, ^263^, ^264^, ^265^ ^]^ The primary objectives of superconducting spintronics are i) colossal magnetoresistance effects by magnetic exchange field,^[^ ^266^, ^267^ ^]^ ii) longer lifetimes of spin‐polarized quasiparticles injected into superconductors than into normal metals,^[^ ^268^ ^]^ iii) switching superconductivity (on/off) via spin injection,^[^ ^267^, ^269^ ^]^ and iv) pure spin‐polarized supercurrents in a spin‐valve configuration, i.e., FM/superconductor/FM.^[^ ^4^, ^268^, ^270^ ^]^ Several experimental and theoretical advances have been achieved for these objectives, such as the Josephson effect in superconductor/FM/superconductor junctions, transition temperature modulation in superconducting spin valves and the generation of triplet pairing states.^[^ ^258^ ^]^ However, the direct measurement of triplet spin transport (generation of spin‐polarized supercurrent in superconductors) has not been achieved yet. Recently, spin currents carried by spin‐triplet pairs, i.e., spin‐polarized supercurrents, have been reported in spin‐valve systems covered by spin sink layers with strong SOC (i.e., heavy metal, Pt) (**Figure** **7**a).^[^ ^258^ ^]^ Further theoretical research is required to establish the details of the spin‐transport process toward superconducting spintronics.

In addition to the generation of pure spin supercurrent, new hybridization of FMs and superconducting orders, which is the interaction of fluxon (Abrikosov vortices) with a static magnetic order (spin density wave, magnon),^[^ ^63^, ^259^ ^]^ has recently been reported. The magnon dispersion was strongly modified via screening currents in the Meissner phase. Unlike the general function of the FM tunnel barriers, this interaction is explained in terms of the modification of the FM properties by superconductivity. More recent results of magnon spectra demonstrate clear evidence of the magnon–fluxon interaction (Figure 7b). The vortex lattice (fluxon) acts as a reconfigurable magnonic crystal, which results in the formation of bandgaps in the magnon spectrum (left panel of Figure 7b). Right panel of figure 7b shows bandgaps in the spin‐wave transmission spectra for a series of out‐of‐plane magnetic field components *µ*_0_ *H*_⊥_(top) and magnetic field dependence of the vortex lattice parameter *a*_VL_(*H*_⊥_) = (2*Φ*_0_/√3*H*_VL_)^1/2^ (bottom). It indicates that bandgaps are affected by the motion of the vortex lattice due to the Doppler effect, resulting in tunable magnons that also facilitate a direct electrical characterization of the vortex flow. This suggests tunable spin‐wave devices and electrical detection of vortex motion with high precision. Hence, this new hybridization of magnons and vortices may be an unexplored opportunity for tuning the magnons of FM media for magnonics using superconductivity.

### 8.2 Superconductivity‐Mediated Quasiparticles toward Quantum Computing

Majorana fermions, first proposed in 1937 are exotic but putative charge‐neutral particles, and they are their own antiparticles unlike Dirac fermions.^[^ ^255^, ^271^ ^]^ Recently, the realization and detection of Majorana fermions in condensed matter as quasiparticles has attracted tremendous interest because of their fundamental novelty and their potential for use as qubits for fault‐tolerant topological quantum computing, aided by their non‐Abelian statistics.^[^ ^254^, ^255^, ^256^ ^]^ Some studies have manipulated the Majorana chain in 1D topological superconductors.^[^ ^272^, ^273^, ^274^ ^]^ The signature of edge‐bound Majorana fermions was detected in an FM atomic chain on a superconductor using spin‐polarized STM (Section 5).^[^ ^66^, ^275^ ^]^ A possible approach to generate the Majorana zero mode is the proximity effect, which consists of ferromagnetically ordered magnetic elements (Zeeman or ferromagnetic exchange interaction) and strong SOC at the surface of the superconductor to engineer topological superconductivity. However, this approach requires extremely clean interfaces, long coherence length and strong SOC for the protection of a Majorana zero mode.

An alternative system approach to realizing Majorana fermions are the helical edge modes, which occur in some large spin–orbit systems (Figure 7c).^[^ ^209^, ^276^ ^]^ A schematic diagram (top left of Figure 7c) shows a hexagonal bismuth bilayer island sitting on the surface of a Bi(111) thin film on superconducting Nb(110) substrate and exhibiting topological helical states on every other edge. ∆_SC_ is a topological superconducting gap induced into helical states. ∆_H_ is a magnetic hybridization gap opened by attaching a ferromagnetic cluster to the bilayer edge. A Majorana zero mode is localized at the mass domain wall realized at the cluster‐helical edge state interface. A sharp zero bias peak (ZBP) within the pairing gap along the Bi bilayer and individual point spectra a localized ZBP at the interface. A magnetic field is not necessary to induce Majorana zero modes that could provide better protection than existing schemes for both disorder and thermal excitation of quasiparticles. This approach of localizing Majorana zero modes in topologically protected helical edge channels may also be realized using vdW heterostructures (top left panel of Figure 7d).^[^ ^209^, ^277^, ^278^, ^279^ ^]^ The realization of Majorana zero modes within the topological edge states can be extended to other 2D or 3D higher‐order TIs such as Fe‐based superconductor.^[^ ^276^, ^280^ ^]^

Another proposed system to generate 2D Majorana fermions includes the *ν* = 5/2 quantum Hall state,^[^ ^281^ ^]^ Moore–Read type state (fractional quantum Hall effect),^[^ ^282^ ^]^ strong SOC semiconductor–superconductor heterostructures^[^ ^283^, ^284^ ^]^ and 2D spinless superconductors with *p_x_ + ip_y_* pairings.^[^ ^285^ ^]^ A more feasible system has been proposed using a 2D topological superconductor formed at the interface between a 3D TI and a conventional s‐wave superconductor, in which Majorana zero modes are expected to be localized in the cores of Abrikosov vortices.^[^ ^5^ ^]^ Magnetic Abrikosov vortices have been probed using scanning tunneling spectroscopy (zero‐bias d*I*/d*V* maps) in the heterostructure of three quintuple layers Bi_2_Te_3_ and NbSe_2_ at 0.4 K and 0.75 T (bottom left panel of Figure 7d). The vortices in heterostructure exhibit a highly ordered hexagonal lattice, similar to those observed on the clean NbSe_2_ surface. It indicates the artificial TS. Abrikosov vortices and bound states inside the vortex core include the predicted Majorana fermion at zero bias. Despite the significant efforts and success in generating topological superconductors, there is still insufficient evidence on 2D Majorana fermions in topological superconductors. Most current experimental evidence for Majorana zero mode relied on scanning tunneling spectroscopy (STS) measurements. However, STS is insufficient to distinguish the Majorana zero mode from other low energy bound states, i.e., Andreev or Caroli‐de Gennes bound states, in the vortex core. These excitations merge together, which results in a single peak cantered at zero energy with certain broadening width in an STS spectrum.^[^ ^286^ ^]^ Hence, a “smoking gun” detection of a Majorana zero mode is necessary.

In addition to Majorana fermions, another interesting quasiparticle is theoretically predicted to be induced in a ferromagnet‐superconductor heterostructure, i.e., magnetic skyrmions (right panels of Figure 7d).^[^ ^257^, ^262^ ^]^ Numerical simulations and analytic arguments reveal broader possibilities for manipulating the skyrmion–vortex dynamic correlations in hybrid systems.^[^ ^257^ ^]^

### 8.3 Challenges

Majorana fermions in topological superconductors that obey a new type of quantum statistics, distinct from the Bose and Fermi statistics, can be applied in topological quantum computation. While some remarkable developments have been achieved, experimental observations of Majorana fermions have been limited to static quasiparticles and localized or bound states. Evidence of Majorana quasiparticles that propagate along the boundaries or walls between different domains in superconductors is still not available. The experimental realization of Majorana fermions in 2D for future applications to topological quantum computation is still an open challenge. Therefore, the proximity‐induced superconductivity in semiconductors or semimetals, such as MoS_2_ and WTe_2_, with large SOCs could be an alternative.^[^ ^19^, ^139^, ^287^, ^288^ ^]^

## 9 Summary, Challenges, and Perspectives

### 9.1 Summary of Multifunctional Proximity Effects in 2D vdW Heterostructures

Here, we briefly describe the status of devices of 3D non‐vdW materials and specify how proximity effects can be used in next‐generation spintronic devices. Traditional FETs and complementary metal‐oxide semiconductors (CMOSs) have been successfully scaled to nanoscale dimensions for high logic/memory performance.^[^ ^76^, ^289^, ^290^, ^291^ ^]^ Spintronic devices based on the manipulation and read‐out of charge‐carrier‐spin have been of primary interest for low‐power electronics, utilizing the spin degree of freedom (Nobel Prize in Physics, 2007).^[^ ^43^, ^238^, ^292^, ^293^, ^294^ ^]^ Since the discovery of GMR in the 1980s,^[^ ^237^ ^]^ spintronic memory devices have advanced rapidly.^[^ ^43^, ^292^, ^293^, ^294^ ^]^ Recently, a TMR ratio of 124% was achieved for magnetic tunnel junctions at room temperature.^[^ ^52^, ^294^, ^295^ ^]^ Despite these remarkable developments, the expansion of big data and the internet necessitates a continuous demand for improved data storage and processing with low power consumption.^[^ ^108^, ^296^ ^]^ The reduction in the size of FETs accelerates the rate of dissipation of heat generated by static power and current leakage and requires advanced fabrication facilities that increase the cost per chip.^[^ ^76^, ^108^ ^]^ The practical development of low‐power spintronic devices requires enhanced conversion efficiency between spin and charge signals.^[^ ^43^, ^73^, ^107^, ^108^, ^294^ ^]^ The control of spin, such as spin–charge conversion, spin transport, and spin manipulation, is the basis of 2D spintronic device operations.

Several challenges hinder the development of electric currents and/or fields with low energy consumption, nonvolatile magnetic memories with ultrafast responses, and innovative methods of generating spin currents/spin polarization. First, it is necessary to explore new low‐dimensional materials and the precise control of their properties beyond 3D CMOS technologies,^[^ ^108^ ^]^ such as 2D vdW layered magnetic (including AFM) materials and topological insulator. Second, the effective operation of FETs requires efficient electrostatic coupling between the electric field induced by the gate bias and channel. Third, effective control of spin currents, spin transport, and spin structures must be realized. In addition, for the delivery of spin information over long distances, spin channels with long spin lifetimes and long spin diffusion lengths should be considered, as the spin‐dependent chemical potential decays exponentially through spin relaxation.^[^ ^43^, ^107^ ^]^ This can be achieved using magnetic proximity with various materials; for example, spin‐momentum locking using TIs, spin‐wave dynamics (magnonics) using AFM or superconductors, spin‐orbitronics such as the spin Hall effect and spin–orbit torque induced using metal or strong SOC materials and current‐induced domain wall motion, including chiral magnetism or skyrmions (DMI). Finally, gate‐tunable FM layers in 2D vdW materials at room temperature are vital for magnetoelectric and magneto‐optic applications,^[^ ^297^ ^]^ in which the magnetic proximity effect could be of significant benefit, i.e., heterostructures with other magnetic materials.^[^ ^90^, ^93^, ^94^, ^113^, ^298^ ^]^

### 9.2 Challenges and Perspectives

In this review, we have explored the magnetic proximity effect and subsequent exotic physics to describe the interfacial effects occurring in 2D material heterostructures. The aim of this review was to explore how to control and enhance the functionalities of 2D vdW layered materials using heterostructures with a magnetic layer for application in next‐generation electronic and spintronic devices. 2D materials and their heterostructures have significant potential for fundamental study and actual applications because of the vast playground offered by their multifunctionalities through generation/confinement/transport of charges, excitons, photons, and phonons of single‐atom thickness. Despite the extraordinary potential and tremendous progress, improving the performance of spin current, extending lifetimes, enhancing the operating temperature and understanding the interfacial effects remain a challenge. Many of these challenges can be resolved by manipulating the magnetic proximity effect in heterostructures. However, for actual applications of spintronics, valleytronics, and quantum computing, additional approaches should be considered to address these limitations.

We briefly recap the challenges of the proximity effect. In sensors and data storage applications, electrically tunable magnetism at room temperature is desired. Sizable valley splitting, valley polarization with high efficiency and longer lifetimes require a strong magnetic exchange field that can be induced by FM layers with a clean surface and high *T*_C_. TMRs with high efficiency and a strong exchange bias effect beyond room temperature are preferred for spintronic devices such as hard drives and MRAMs.^[^ ^201^ ^]^ In addition, the generation/detection of spin current and noncollinear spin structures, fast domain wall motion for racetrack‐type magnetic memories, logic devices, and the novel magnetoresistance effect are still challenging.^[^ ^172^, ^299^ ^]^ For future applications of topological quantum computation, Majorana fermions in topological superconductors should be realized and detected experimentally.

**Figure 8:**
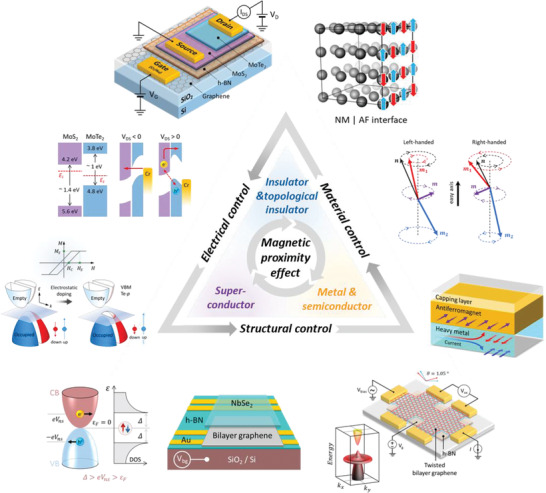
Schematic diagram of the perspectives of interplay between proximity effects and controllable effects. Electrostatic gating to control the band structure or transfer charge/spin.^[^ ^97^, ^304^, ^322^ ^]^ Structural perturbation induced by stacking or twisting or strain.^[^ ^131^ ^]^ Survey of materials and spin textures.^[^ ^62^, ^323^ ^]^

To overcome these challenges, we suggest engineering the synergistic effects caused by the interplay between the interfacial intrinsic proximity effects and three additional approaches (**Figure** **8**): i) tuning the band structure through electrostatic gating, ii) structural perturbation mainly induced by stacking sequence and subsequent bandgap engineering, and iii) AFM spin textures and dynamics as a substitute for 2D FM materials. In addition to these challenges, instability of 2D vdW layered materials in air is an extrinsic challenge to address for practical applications. These challenges and resolutions have been explored in several recent reviews.^[^ ^167^, ^300^ ^]^

We first consider the electrostatic gating effect to tune the functionalities of the materials, including the band structure. Electrostatic gating, which is the basis of modern electronic technology, can be understood in terms of FETs for conventional electronics.^[^ ^7^ ^]^ 2D materials with atomically flat interfaces and atomic‐scale thicknesses are suitable candidates for electronic structure (i.e., electronic bandgap and exciton binding energy) tuning through displacement fields across the device. Alternatively, one can also use electrostatic gating to modulate the carrier density. Charge transfer is restricted by strong interfacial coupling, which requires high‐quality matched interfaces between component materials. In contrast to the conventional heterostructure of 3D materials with a depletion region, 2D vdW heterostructures reduce the number of charge traps and/or scattering and consequently facilitate ultrafast charge transfer despite weak interfacial binding.^[^ ^298^ ^]^ This electrical approach has been applied in recent advances in 2D material systems, such as electric‐field‐induced superconductivity or metal–insulator transitions.^[^ ^301^, ^302^ ^]^ It can be conducted to control the spin current and spin lifetime of a 2D system.^[^ ^303^ ^]^ In asymmetric vdW heterostructures, charge‐carrier injection can be achieved and switched between tunneling and thermal activation depending on the bias condition. Consequently, a high on/off current and rectifying ratio can be achieved.^[^ ^304^ ^]^

Next, we consider structural perturbations such as the stacking configuration, periodicity, and tilt angle. The designable stacking periodicity and twisting angle can realize a tunable “knob” for controlling and creating functionalities of 2D materials, such as adjusting the magnetic state from AFM to FM/FiM^[^ ^305^ ^]^ or superconductivity in graphene combined with electrostatic gating.^[^ ^131^ ^]^ 2D vdW materials have superior mechanical flexibility, which is relevant for strain engineering and can be applied to tune the electronic band structure, for example, by tuning from a semiconductor to a metal.^[^ ^298^ ^]^ Recently, the Moiré pattern, which is an interference pattern^[^ ^306^, ^307^ ^]^ caused by the rotation between any two regular layers, has attracted immense interest because of its ability to control electronic properties. Moiré patterns exist not only in identical layered materials but also in overlapping heterostructures with minor lattice mismatches.^[^ ^308^ ^]^ As a result of the weak periodic potential imparted by the Moiré pattern, the electronic band structure can be radically altered. According to theoretical calculations, a Moiré pattern can modulate the magnetic proximity effect and manifest miniband spin splitting.^[^ ^309^ ^]^ Another interesting theoretical prediction is the formation of skyrmions in the Moiré of vdW magnets^[^ ^252^ ^]^ because of the small lattice mismatch between the AFM substrate, such as layered manganese chalcogen phosphates, MnPX_3_, X = S, Se, T and the FM monolayer including chromium trihalides, CrX_3_, X = Cl, Br, and I. Moreover, the twisted Moiré is another possible approach to induce magnetization textures and skyrmions in 2D magnets.^[^ ^310^ ^]^

Finally, we consider the material science challenges. Here, we discuss the ability of AFM materials to induce nonrelativistic (i.e., SOC‐unrelated) GMR and spin‐transfer torque phenomena.^[^ ^221^, ^311^, ^312^ ^]^ AFM materials can generate, detect, and transmit spin currents^[^ ^221^ ^]^ and they have numerous advantages over FM materials.^[^ ^313^ ^]^ Generally, it is difficult to control AFM materials through external magnetic fields owing to zero‐net magnetization. Unlike FM‐based devices that are vulnerable to reorientation or erasure by external magnetic fields, the data stored on AFM‐based devices are invisible to external magnetic probes. In addition, AFM materials demonstrate ultrafast spin dynamics with a broad range of materials, such as metals, semiconductors, or insulators, with *T*_N_ above room temperature.^[^ ^314^ ^]^ The relatively high *T*_N_ of AFM materials facilitates the induction of the FM order with a much higher *T*_C_ in adjacent materials. A recent study demonstrated the interfacial spin texture and enhanced *T*_C_ in a magnetically doped TI interface.^[^ ^23^, ^313^ ^]^ Insulating AFM substrates (i.e., AFM insulators) are better candidates for inducing magnetic orders in the TI layers. In terms of domain wall motion, the velocity of the domain wall is limited by the magnon velocity, which is significantly larger than that of a typical magnon.^[^ ^315^, ^316^ ^]^ The internal exchange torque in the AFM layers is several orders of magnitude larger than any driving torque. Therefore, the advantage of AFM layers is the electrical switching of AFM domains through the current‐induced Néel spin–orbit torque. AFM layers can convert electronic‐spin current into magnonic‐spin current, which can generate a magnon flux with nonzero spin, thereby enhancing spin injection and pumping. In terms of device design, it is essential to examine how these fluctuations vary in proximity to capping layers (materials and thickness).^[^ ^317^, ^318^, ^319^ ^]^ To increase the magnitude of the spin–orbit torque, numerous studies have been conducted for different multilayers consisting of heavy metals, FM layers, and capping layers, including materials and thicknesses. In particular, capping layer materials determine the strength of spin–orbit torque efficiency by reflecting and absorbing the induced spin currents.^[^ ^318^ ^]^ According to recent theoretical calculations, capping layers with a long spin diffusion length and high resistivity should enhance the spin Hall torque.^[^ ^319^ ^]^

## Conflict of Interest

The authors declare no conflict of interest.
